# Effect of transcription factor resource sharing on gene expression noise

**DOI:** 10.1371/journal.pcbi.1005491

**Published:** 2017-04-17

**Authors:** Dipjyoti Das, Supravat Dey, Robert C. Brewster, Sandeep Choubey

**Affiliations:** 1 Department of Molecular, Cellular and Developmental Biology, Yale University, New Haven, Connecticut, United States of America; 2 Laboratoire Charles Coulomb, Université de Montpellier and CNRS, Montpellier, France; 3 Program in Systems Biology, University of Massachusetts Medical School, Worcester, Massachusetts, United States of America; 4 Department of Microbiology and Physiological Systems, University of Massachusetts Medical School, Worcester, Massachusetts, United States of America; 5 FAS Center for Systems Biology, Harvard University, Cambridge, Massachusetts, United States of America; 6 Department of Molecular and Cellular Biology, Harvard University, Cambridge, Massachusetts, United States of America; Center for Genomic Regulation, SPAIN

## Abstract

Gene expression is intrinsically a stochastic (noisy) process with important implications for cellular functions. Deciphering the underlying mechanisms of gene expression noise remains one of the key challenges of regulatory biology. Theoretical models of transcription often incorporate the kinetics of how transcription factors (TFs) interact with a single promoter to impact gene expression noise. However, inside single cells multiple identical gene copies as well as additional binding sites can compete for a limiting pool of TFs. Here we develop a simple kinetic model of transcription, which explicitly incorporates this interplay between TF copy number and its binding sites. We show that TF sharing enhances noise in mRNA distribution across an isogenic population of cells. Moreover, when a single gene copy shares it’s TFs with multiple competitor sites, the mRNA variance as a function of the mean remains unaltered by their presence. Hence, all the data for variance as a function of mean expression collapse onto a single master curve independent of the strength and number of competitor sites. However, this result does not hold true when the competition stems from multiple copies of the same gene. Therefore, although previous studies showed that the mean expression follows a universal master curve, our findings suggest that different scenarios of competition bear distinct signatures at the level of variance. Intriguingly, the introduction of competitor sites can transform a unimodal mRNA distribution into a multimodal distribution. These results demonstrate the impact of limited availability of TF resource on the regulation of noise in gene expression.

## Author summary

Genetically identical cells, even when they are exposed to the same environmental conditions, display incredible diversity. Gene expression noise is attributed to be a key source of this phenotypic diversity. Transcriptional dynamics is a dominant source of expression noise. Although scores of theoretical and experimental studies have explored how noise is regulated at the level of transcription, most of them focus on the gene specific, cis regulatory elements, such as the number of transcription factor (TF) binding sites, their binding strength, etc. However, how the global properties of transcription, such as the limited availability of TFs impact noise in gene expression remains rather elusive. Here we build a theoretical model that incorporates the effect of limiting TF pool on gene expression noise. We find that competition between genes for TFs leads to enhanced variability in mRNA copy number across an isogenic population. Moreover, for gene copies sharing TFs with other competitor sites, mRNA variance as a function of the mean shows distinct imprints for one gene copy and multiple gene copies respectively. This stands in sharp contrast to the universal behavior found in mean expression irrespective of the different scenarios of competition. An interesting feature of competition is that introduction of competitor sites can transform a unimodal mRNA distribution into a multimodal distribution, which could lead to phenotypic variability.

## Introduction

Every living organism regulates gene expression through the action of transcription factors (TFs), enabling the cell to respond to intra-cellular and environmental cues [1]. The binding and unbinding of both RNAP molecules and the TFs (DNA binding proteins that abet or hinder RNAP binding) to the promoter [2–4], are inherently stochastic processes and this stochasticity is manifested in the output of gene expression [5–11]. Consequently, at the single cell level, the numbers of mRNA and protein molecules fluctuate in time and across populations. Such fluctuations in expression can be detrimental to cell fitness [12–14] and the development of multicellular organisms [15]. On the contrary, noisy expression can benefit a population of genetically identical cells by creating phenotypic heterogeneity [16–21, 21–30]. This raises the question of how noise in gene expression is regulated [31, 32]. Over the past decade or so, theorists have sought to unravel how gene expression noise is regulated [33–40]. Meanwhile, experimentalists have measured noise at both the mRNA and protein level in prokaryotes [41–45] and eukaryotes [46–48], in order to systematically test the predictions of these models, and refine our understanding.

Models of transcription quintessentially hinge on the details of the promoter architecture such as the number and affinity of TF binding sites and relative binding positions on the gene [39, 49–54]. Most of these theoretical models implicitly assume that the number of TFs is in excess with respect to the number of its binding sites in the cell. However, inside the cell this assumption often breaks down. For example, TFs get shared by multiple gene copies, in highly replicated viral DNA genes [55], genes expressed on plasmids [56], and in multiple identical copies on the chromosome [57–61]. Furthermore, the majority of TFs are entrusted with the regulation of multiple genes; for instance, cAMP receptor protein (CRP) is reported to have nearly 400 binding sites per *E. coli* genome copy [62, 63]. It is therefore crucial to predict gene expression noise due to limited availability of TF resource, in order to dissect how the sharing of TFs among multiple genes contributes to noise.

Some studies have explored the interplay of TF sharing between genes and other competing binding sites in different scenarios [44, 64–72]. For instance, in a recent study, Rydenfelt et al. [73] have theoretically explored the effect of TF sharing on the mean level of gene expression, which has since been verified experimentally [65, 67]. However, few studies have systematically explored how competition, for TF binding influences gene expression noise [68–71]. In one such study, it was found that in the context of a single auto-regulated gene copy, the addition of decoy binding sites can lead to bimodal protein distributions in an isogenic population. Although these studies provide useful insights, nevertheless a general theoretical framework of understanding how TF resource sharing affects noise in gene expression remains in its infancy. The goal of this study is to develop such a framework.

**Fig 1:**
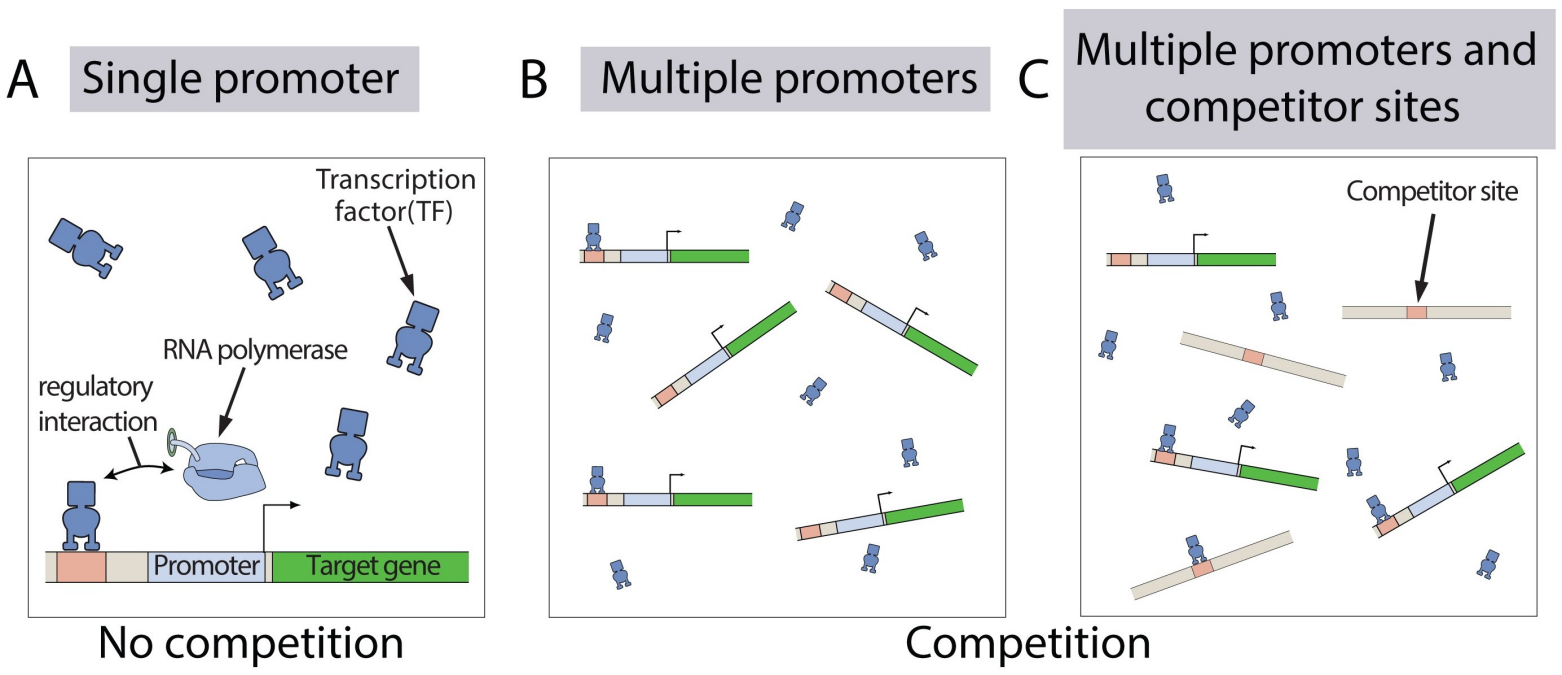
Different scenarios of competition for a pool of TFs among promoters and other binding sites. (A) A single promoter copy in isolation without the presence of any other TF binding sites. (B) Multiple copies of identical promoters competing with each other for the TFs. (C) Promoters compete with other competitor sites for the TFs. Competitor sites can correspond to ‘decoy sites’ or promoters driving expression of other gene species. Therefore, although the competitor sites bind to the TFs, they do not regulate the production of mRNA molecules from the target promoters.

Here we examine a simple model of transcription, where a number of TFs are shared between a comparable number of gene copies and other competitor sites. We explicitly envisage two different scenarios of competition, demonstrated schematically in Fig 1: (i) multiple identical promoters sharing their TFs (Fig 1B), and (ii) promoters sharing their TFs with other competitor sites (Fig 1C). These competitor sites can represent ‘decoy sites’ or promoters driving expression of other gene species. We find that competition enhances noise across an isogenic population, i.e. the Fano factor (defined as the variance divided by the mean) of the mRNA distribution shows a peak when the TF number becomes comparable to the promoter number. Interestingly when a single promoter copy shares TFs with multiple competitor sites in a cell, we find that there exists a master curve for the mRNA variance as a function of its mean that is independent of the binding, unbinding rates and the number of competitor sites. However, this result does not hold when a number of promoter copies compete for a pool of TFs. In other words, these different scenarios of TF competition bear distinct signatures at the variance level. This finding stands in sharp contrast to the behavior at the mean level [67], which follows a universal master curve irrespective of the nature of competition. We further find that a unimodal mRNA distribution without any competitor sites can be transformed into a multimodal distribution when competitor sites are introduced. For such a multimodal distribution, different modes arise and diminish as the number of competitor sites is systematically tuned. While our model predictions are consistent with recent experimental [67] and theoretical [73] studies at the mean level of expression, we also find that TF competition can have a complex impact on the noise in expression which results in multimodality and a scaling with mean expression level that depends on the source of the competition.

## Results

### Model

In order to investigate how TF sharing affects cell-to-cell variability at the mRNA level across a population of genetically identical cells, we examine a simple model of transcription, where a number of TFs are shared between a comparable number of identical promoter copies and competitor sites (see Fig 1B and 1C). Moreover, as case studies, we consider two regulatory motifs of transcription in *E. coli* [74]: 1) a promoter containing a single binding site where a repressor molecule can bind and hinder transcription, and 2) a promoter consisting of a single binding site where an activator molecule can bind and increase the rate of transcription. These regulatory architectures have been extensively explored in different studies, using the implicit assumption that TFs are in abundance [38, 64, 75–79].

**Fig 2:**
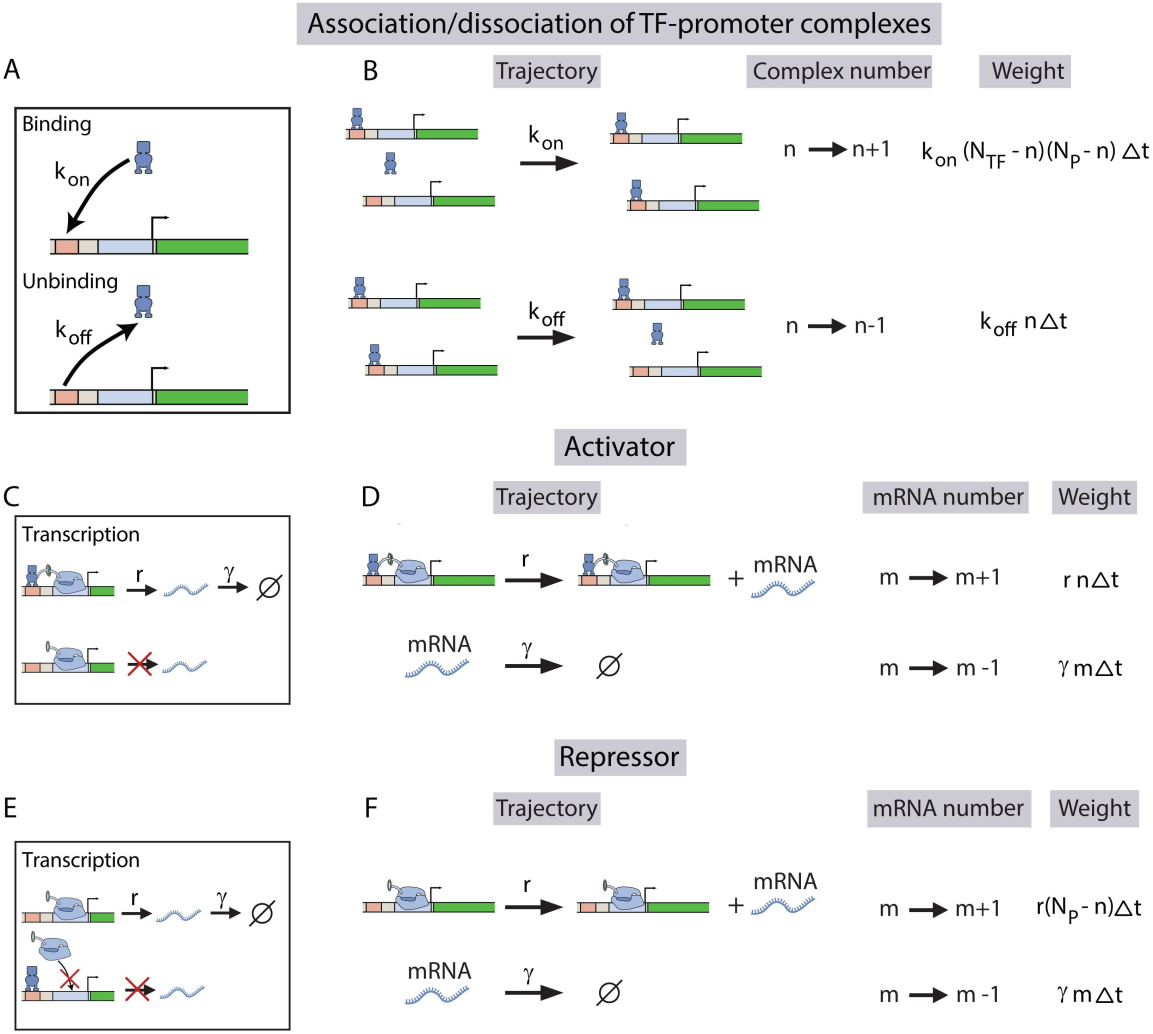
A kinetic model of transcription, incorporating the TFs’ binding and unbinding to multiple competing promoters. (A) The rate of binding of a TF to a promoter is *k*_on_ and the rate of unbinding of the TF from the promoter is *k*_off_. (B) List of possible stochastic transitions leading to either formation or dissociation of ‘TF-promoter’ complexes, and their respective statistical weights. The weights represent the probability that each transition will occur during a time interval, Δ*t*. TF copy number, promoter copy number, and the instantaneous number of complexes are denoted by *N*_TF_, *N*_P_ and *n* respectively. (C) While an activator is bound to a promoter, it promotes the binding of RNAP molecules to the promoter, which subsequently leads to the production of an mRNA molecule at a rate *r*. Each mRNA molecule then decays at a rate *γ*. Here, *m* denotes mRNA copy number. The basal transcription rate, when the activator is not bound to the promoter, is assumed to be zero (see S1 Text for a more general model for activators with a nonzero basal rate). (D) List of stochastic transitions leading to changes in mRNA copy number by the action of activators and their respective weights. (E) When a repressor is bound to a promoter, it hinders RNAP binding to the promoter, subsequently blocking mRNA production. However when a promoter is not bound to any repressor molecule, it produces mRNA molecules at a rate *r*, which again subsequently decay at a rate *γ*. (F) List of stochastic transitions leading to changes in mRNA copy number by the action of repressors, with respective weights.

We construct a general model of transcription, that explicitly incorporates the interplay between TF copy number and its binding sites. To elucidate the model, we first consider a number of identical promoters sharing a pool of TFs, as shown in Fig 2A in the absence of any competitor sites. We can easily incorporate the presence of competitor sites in our model, as shown in the later sections. For the sake of generality we discuss the process of TF binding and unbinding generically without regard to their regulatory functions; later we will outline the influence of activators and repressors separately. Let us consider *N*_TF_ number of TFs being shared by *N*_P_ number of promoters, where each promoter contains a single TF binding site. A TF binds to a promoter at a rate *k*_on_, and it unbinds from it at a rate *k*_off_ (see Fig 2A). For mathematical convenience we view the process of TF binding to a promoter as the formation of a ‘TF-promoter’ complex, and the unbinding of the TF as dissociation of the complex [80]. Consequently, complex formation rate per TF per promoter is *k*_on_, while dissociation rate per complex is *k*_off_ (see Fig 2B).

The number of TF-promoter complexes determines the transcriptional output. In the case of activators, mRNA production takes place when the activator is bound to a promoter (see Fig 2C). Each of the activator-promoter complexes produces mRNA molecules at a rate *r*, which then subsequently degrades at a rate *γ*. Here we have assumed for simplicity that the basal transcription rate, when the promoter is not bound to an activator, is zero, as it is negligible for many activator regulated genes [62]. However, all our results still qualitatively hold even when we incorporate a non-zero basal rate of transcription (see Fig D in S1 Text). On the other hand, since repressor binding to a promoter hinders the attachment of RNA polymerases to the promoter, each promoter produces mRNA molecules at a rate *r*, only when it is not bound to a repressor, as shown in Fig 2E. In the limit of one promoter copy, our model reduces to the well known ON-OFF model [75, 81–84], where the promoter switches between an active and an inactive state, and transcription can ensue only in the active state.

The above processes can be depicted as trajectories in the space of TF-promoter complex number and mRNA copy number, as shown in Fig 2B, 2D and 2F. By employing a stochastic framework, we monitor the time evolution of two random variables: the instantaneous number of TF-promoter complexes, *n*(*t*) and the instantaneous number of mRNA molecules, *m*(*t*). Evidently for activators, the instantaneous mRNA production rate is proportional to the number of complexes (*n*); while for repressors, the mRNA production rate is proportional to the number of unbound promoters (*N*_P_ − *n*) (compare Fig 2D and 2F). Using the master equation approach, we write down the time-evolution equation for the joint probability distribution *P*(*n*, *m*, *t*) of having *n* complexes and *m* mRNA molecules at a time *t*. For instance, the master equation for activators is given by

$$\begin{array}{ll} \frac{dP\left(n,m,t\right)}{dt}= & k_{\text{on}}\left(N_{\text{TF}}-n+1\right)\left(N_{\text{P}}-n+1\right)P\left(n-1,m,t\right)+k_{\text{off}}\left(n+1\right)P\left(n+1,m,t\right)\\ & +rnP\left(n,m-1,t\right)+\gamma \left(m+1\right)P\left(n,m+1,t\right)\\ & -\left[k_{\text{on}}\left(N_{\text{TF}}-n\right)\left(N_{\text{P}}-n\right)+k_{\text{off}}n+rn+\gamma m\right]P\left(n,m,t\right). \end{array}$$ (1)

The above equation is built by counting all possible steps that lead to either a change in complex number (*n*) or in mRNA copy number (*m*) by one (see trajectories in Fig 2B and 2D). Similarly, we could also write the master equation for repressors by combining the trajectories depicted in Fig 2B and 2F (see S1 Text for details). In principle, we can obtain the relevant moments of the mRNA distribution from these master equations. However, exact expressions of mean (〈*m*〉) and variance (var(*m*)) of the mRNA distribution can only be obtained for the simplest case of one promoter. For the general case of multiple promoters, deriving exact closed-from expressions for the moments is challenging, because the system of equations for the moments do not close. Nonetheless, we can obtain approximate analytical solutions (see S1 Text for details).

Since we do not have exact analytical solutions for the moments of the mRNA distribution, we simulate the kinetic processes defined above using the Gillespie algorithm [85]. This allows us to numerically generate multiple time-traces of the random numbers *n* and *m*, using realistic values of the kinetic rates suitable for *E. coli* promoters (see Table 1). From these different time traces, we calculate the values of the mean and variance of the steady state mRNA distribution. The approximate analytical solutions match reasonably well with our simulation data (see Fig A in S1 Text). While there is a systematic deviation which results from ignoring higher order terms (beyond second order), these closed form relations can be useful in understanding the qualitative behaviors of these curves, which the analytical solutions do capture (see S1 Text for details). Below we summarize our main results, obtained from the Gillespie simulations.

**Table 1:** Kinetic rates that are used in simulations.

Kinetic processes	Symbols of rates	Values of rates
Activator/repressor binding	*k*_on_	0.0027*s*^−1^ (per TF per promoter) [42]
Activator/repressor unbinding	*k*_off_	0.0023*s*^−1^ (per TF-promoter complex) [86]
mRNA production (transcription)	*r*	0.33*s*^−1^ (per ‘activator-bound’ or ‘repressor-free’ promoter) [87]
mRNA decay	*γ*	0.011*s*^−1^ (per mRNA) [41]

### Multiple identical promoters competing for a pool of TFs

Armed with the framework established above, we first investigate the scenario when multiple identical promoter copies compete for a pool of TFs (as shown in Fig 1B). Our model predicts the effect of TF sharing on the mean (〈*m*〉) and variance (var(*m*)) of the steady-state mRNA distribution. We shall first discuss the case when the TFs act as activators, and then shift our focus to the case of repressors.

**Fig 3:**
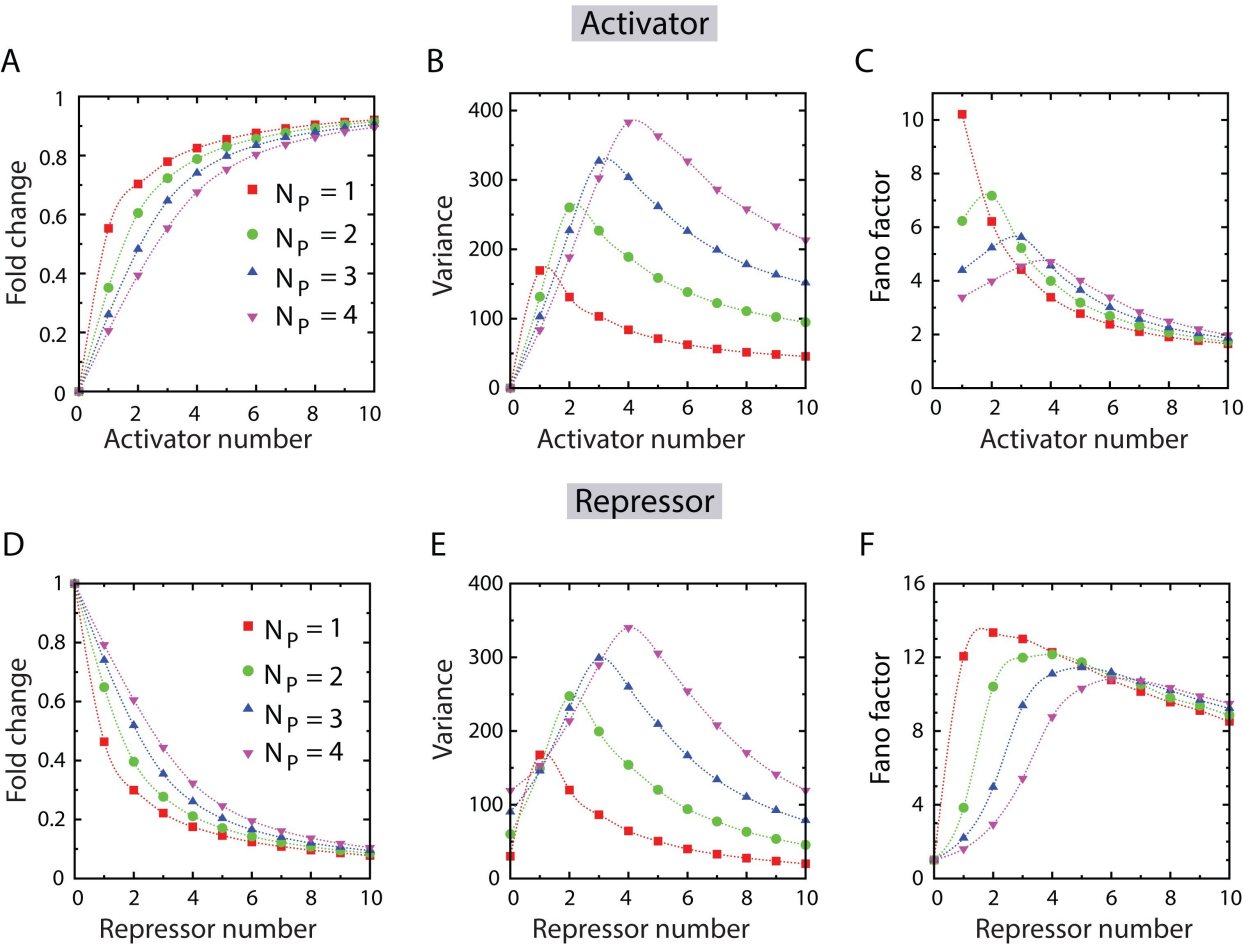
Our model of TF sharing predicts the behavior of the first two moments of steady-state mRNA distribution across an isogenic population. (A) Fold change of the mRNA distribution (defined by 〈*m*〉/(*N*_P_*r*/*γ*)) as a function of activator number. (B) Variance of mRNA distribution (var(*m*)) versus activator number. Note that the variance peaks when the activator number equals the promoter number. (C) Fano factor (defined by var(*m*)/〈*m*〉) also exhibit a peak as a function of activator number, when the activator number equals the promoter number. (D) Fold change of expression as a function of repressor number. (E) Variance of mRNA distribution versus repressor number. Just like activators, the variance peaks when repressor number equals the promoter number. (F) Fano factor versus repressor number, showing peaks when the repressor number becomes comparable, but slightly higher than the promoter number. In all the plots, symbols represent data from numerical simulations, which are connected by smooth dashed lines to guide the eyes. The parameter values are as specified in Table 1.

We explore the effect of activator sharing in the fold-change of mean expression, a quantity often measured in bulk assays (see Fig 3A) [67, 73]. Here we define the fold-change as the ratio of the mean expression 〈*m*〉 to its maximum value *N*_*P*_(*r*/*γ*). The mean of the mRNA distribution, increases steadily with activator number, and asymptotically goes towards the value of *N*_P_(*r/γ*) (see Fig F in S1 Text). In the limit of activator copy number being much greater than the promoter copy number, all the promoters essentially remain bound to activators. Accordingly each of these activator-promoter complexes contributes, on average, *r*/*γ* mRNA molecules, resulting in the production of *N*_P_(*r/γ*) mRNA molecules on average, in the steady state. The qualitative behavior of fold-change, which first monotonically increases with activator number and asymptotically approaches one, is in agreement with previous studies [73].

In contrast to the fold-change, variance is a non-monotonic function of the number of activator molecules, which peaks when the number of activators equals the promoter copy-number (see Fig 3B and also see Fig F in S1 Text). The origin of the peak in variance lies in the activator-promoter complex number variability. In fact the variance in complex number also peaks when the promoter copy number equals activator copy number (see Fig B in S1 Text). In other words, when activator number is comparable to promoter copy-number, there is enhanced variability in complex number stemming from the stochastic complex formation and dissociation processes, which subsequently gets reflected at the level of mRNA distribution. On the contrary, if the numbe of activators is either much greater or much less than the promoter number (in the limits of *N*_TF_ ≫ *N*_P_ and *N*_TF_ ⪡ *N*_P_), the effect of competition disappears, as all the promoters remain either bound or unbound to activators. If we consider the example of activator number being much higher than promoter number, all the identical gene copies driven by the promoters will be expressed all the time. This will lead to each gene copy producing mRNA molecules which is Poisson distributed across an isogenic population [54]. Hence the distribution of all the mRNAs produced from all the identical gene copies will be given by the sum of the numbers of mRNA produced from every gene, leading to a reduction in mRNA variability and a sharply peaked distribution. We further show in Fig 3C the effect of activator sharing on the Fano factor (var(*m*)/〈*m*〉). Just like the mRNA variance, the Fano factor also exhibits non-monotonicity as a function of activator number, and peaks when the activator and promoter numbers become equal.

Turning our attention to repressors, we find an analogous result where instead of gene expression being proportional to the number of activator-promoter complexes; it is now proportional to the number of promoters not bound to repressors. As such, we find that the fold-change, defined by 〈*m*〉/(*N*_P_*r*/*γ*), monotonically decreases with increasing repressor number, and asymptotically approaches zero (see Fig 3D). Moreover, the variance is a non-monotonic function of repressor number, and exhibits a sharp peak when the number of repressors equal promoter copy number, as was the case for activators (see Fig 3E). As before the appearance of these peaks can be understood from a corresponding enhancement in complex number variability (see Fig B in S1 Text). Finally, the non-monotonic behavior of Fano factor, found in the case of activators, also persists for repressors, when the Fano factor is plotted as a function of the repressor number (see Fig 3D). However, there is a distinction between the cases of activators and repressors at the level of Fano factor. Unlike the activators, the Fano factor for repressors do not peak exactly when the repressor number equals the promoter number; rather the peaks appear when the repressor number is slightly greater than (although comparable to) the promoter number (see Fig 3D). These behaviors are very robust with respect to changes in the different parameter values associated with the model (for details see the S1 Text, Figs G-I).

In summary, a key prediction of our model is that TF sharing among identical promoters leads to a substantial increase in gene expression noise when TF and promoter copy number become comparable to each other. Sharp peaks arise in mRNA variance and Fano factor as a function of TF copy number, which bears the signature of pronounced competition.

### Promoters sharing TFs with other competitor sites

Inside single cells, TFs often promiscuously bind to different competitor sites [42, 88, 89] such as ‘decoy sites’ or promoters driving expression of other gene species, compelling a promoter of interest to compete with these sites for a limiting pool of TFs (as shown in Fig 1C). Recent bulk studies have theoretically [68, 73] and experimentally [67, 90] studied this scenario inside bacterial cells, by synthetically integrating multiple competitor sites at several chromosomal locations or on plasmids.

**Fig 4:**
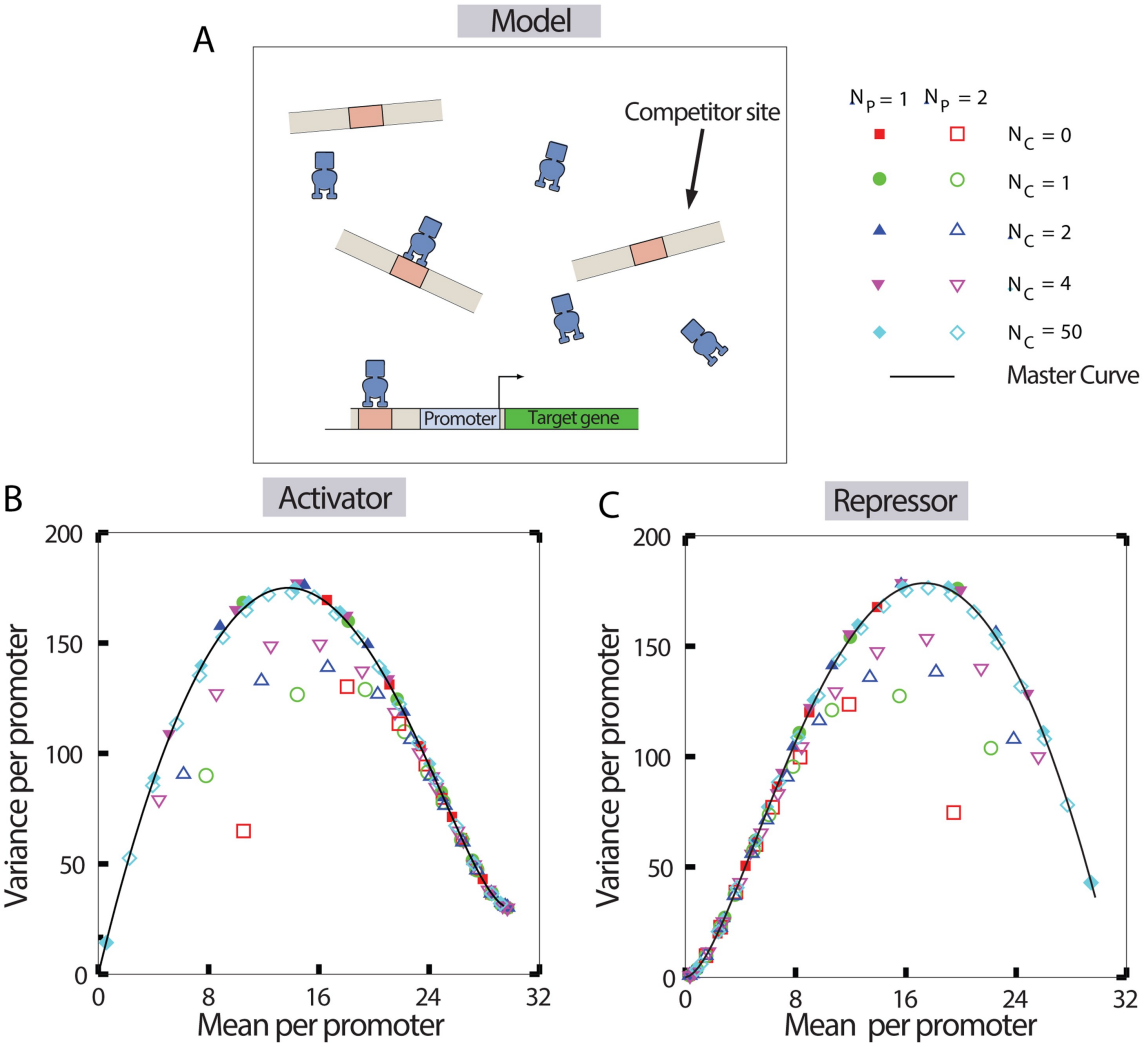
Prediction of mRNA variance as a function of mean, when the promoters share TFs with other competitor sites. (A) Schematic of a single target promoter sharing TFs with multiple competitor sites. (B) The variance of mRNA molecules per promoter (var(*m*)/*N*_P_) versus the mean per promoter (〈*m*〉/*N*_P_), when the TFs act as activators. (C) The mRNA variance per promoter versus mean per promoter when the TFs act as repressors. In both the subfigures B and C, different numerical data points are obtained for various numbers of competitor sites by varying the TF copy number. Note that all the data for a single target promoter collapse onto a master curve (black curve). Data for a single target promoter is shown by closed symbols, and the data for two target promoters are shown by open symbols. The parameters related to the promoters (*k*_on_, *k*_off_, *r*, *γ*) are taken from the Table 1, while the number of competitor sites are systematically varied and listed in the figures. The master curves are predictions from Eq 3.

To explore how this interplay affects the cell-to-cell variability at the mRNA level, we incorporate TF binding to competitor sites, and extend our model as described before to consider *N*_P_ number of promoters competing for *N*_TF_ number of TFs in the presence of *N*_C_ number of competitor sites (shown in Fig 4A). Consequently, every TF can bind either to a promoter or to a competitor site. The rates at which a TF binds to a competitor site and unbinds from it are given by $k_{\text{on}}^{C}$ and $k_{\text{off}}^{C}$ respectively. We first discuss how the competition of a single promoter copy (*N*_P_ = 1) with multiple competitor sites affects the variability at the mRNA level.

A key prediction of this model is that for both activators and repressors, the variance as a function of the mean remains unaltered, regardless of the number and the strength of the competitor sites, as shown in Fig 4B and 4C (also see Fig E in S1 Text). In other words, when we tune the variance as a function of the mean by altering TF number *N*_*TF*_, all the data points for variance, generated from numerical simulations using different parameter values for the number of competitor sites collapse onto a single master curve. This result holds true even for different TF binding/unbinding rates to the competitor sites (please see Fig E in S1 Text). In order to develop an intuition for this result, we employ already existing analytical results involving TF regulating a single promoter copy [37]. In fact, when the number of competitor sites is zero, it is easy to obtain exact analytical expressions for the variance and the mean from the master equation (Eq 1), which are given by,

$$\begin{array}{lll} \langle m\rangle = & \frac{r}{\gamma }\frac{k_{\text{on}}N_{\text{TF}}}{\left(k_{\text{on}}N_{\text{TF}}+k_{\text{off}}\right)} & \\ \text{var}\left(m\right)= & \langle m\rangle \left[1+\frac{k_{\text{off}}}{\left(k_{\text{on}}N_{\text{TF}}+k_{\text{off}}\right)}\frac{r}{\left(\gamma +k_{\text{on}}N_{\text{TF}}+k_{\text{off}}\right)}\right]\; & (\text{for activators}),\\ \langle m\rangle = & \frac{r}{\gamma }\frac{k_{\text{off}}}{\left(k_{\text{on}}N_{\text{TF}}+k_{\text{off}}\right)} & \\ \text{var}\left(m\right)= & \langle m\rangle \left[1+\frac{k_{\text{on}}N_{\text{TF}}}{\left(k_{\text{on}}N_{\text{TF}}+k_{\text{off}}\right)}\frac{r}{\left(\gamma +k_{\text{on}}N_{\text{TF}}+k_{\text{off}}\right)}\right]\; & (\text{for repressors}). \end{array}$$ (2)

It is conceivable from the above analytical expressions, that both the variance and the mean can be expressed as simple functions of an ‘effective’ binding rate of the TFs to the promoter, $k_{\text{on}}^{\text{eff}}=k_{\text{on}}N_{\text{TF}}$ (also see Eq. 14-15 in S1 Text). The presence of the competitor sites essentially modifies the TF pool as seen by the promoter i.e. the effective binding rate of the TFs to the promoter without altering any of the other parameters such as, *k*_off_, *r* and *γ*. Hence we can express the variance as a function of the mean by eliminating the dependence of these functions on the effective binding rate, $k_{\text{on}}^{\text{eff}}$. For both the activators and repressors, we can thus express the variances as functions of the means given by,

$$\begin{array}{lll} \text{var}\left(m\right)= & \langle m\rangle +\frac{\langle m\rangle {\left[\left(r/\gamma \right)-\langle m\rangle \right]}^{2}\gamma }{\gamma \left[\left(r/\gamma \right)-\langle m\rangle \right]+\left(r/\gamma \right)k_{\text{off}}} & \left(\text{for activators}\right),\\ \text{var}\left(m\right)= & \langle m\rangle +\frac{{\langle m\rangle }^{2}\left[\left(r/\gamma \right)-\langle m\rangle \right]\gamma }{\gamma \langle m\rangle +\left(r/\gamma \right)k_{\text{off}}} & \left(\text{for repressors}\right). \end{array}$$ (3)

These are the functional forms of the master curves, which exactly match the collapsed data, as shown in Fig 4B and 4C. In other words, for a single promoter copy, noise at the mRNA level changes only as a function of the mean, irrespective of whether the promoter of interest is competing for a pool of TFs with competitor sites or not. Moreover, this result holds true even when there exists a basal rate of transcription for promoters not bound to activators (see Fig E in S1 Text). This highlights an intriguing prediction for transcriptional noise in the face of competition; the noise depends only on the mean, and it is determined solely by an effective binding rate of the TFs to that promoter, irrespective of the interaction of the TFs with the competitor sites. In fact this result also implies that variance as a function of the mean cannot be used as a signature to distinguish scenarios where a single promoter copy is in competition for a pool of TF with other competitor sites.

Next, we investigate the case of multiple promoter copies competing for a pool of TFs with a number of competitor sites. Our model predicts that the collapse, as observed in the variance as a function of the mean for a single promoter copy does not hold anymore. To explicate this further, we consider the hypothetical situation of two promoter copies, competing with other competitor sites. When we systematically tune the number of competitor sites and using gillespie simulations obtain the variance as a function of the mean, we find that the different curves, defining the variance per promoter as a function of the mean per promoter, no longer collapse onto a single master curve, as shown in Fig 4B and 4C (open symbols). We note that the TF copy number variation, affects mRNA production from both the promoter copies. Hence the numbers of mRNAs produced from the two copies become correlated [73]. Owing to this correlation, the mean and variance cannot be simply expressed in terms of some effective variable, such as *k*_on_*N*_TF_, as before (see Eq. 17 in S1 Text, where nonlinear terms, like $k_{\text{on}}^{2}N_{\text{TF}}^{2}$, appear in the expressions). However, in the limit of the TF copy number or the number of competitor sites becoming much greater than one (i.e. *N*_TF_ ≫ 1 or *N*_C_ ≫ 1), the correlation between the number of mRNAs produced from different promoter copies become negligible. In both these limits the data for variance per promoter versus mean per promoter again fall on the master curve obtained for a single promoter copy, signifying that the promoters behave independently (see Fig 4B and 4C).

### Introduction of competitor sites leads to multimodal mRNA distribution

**Fig 5:**
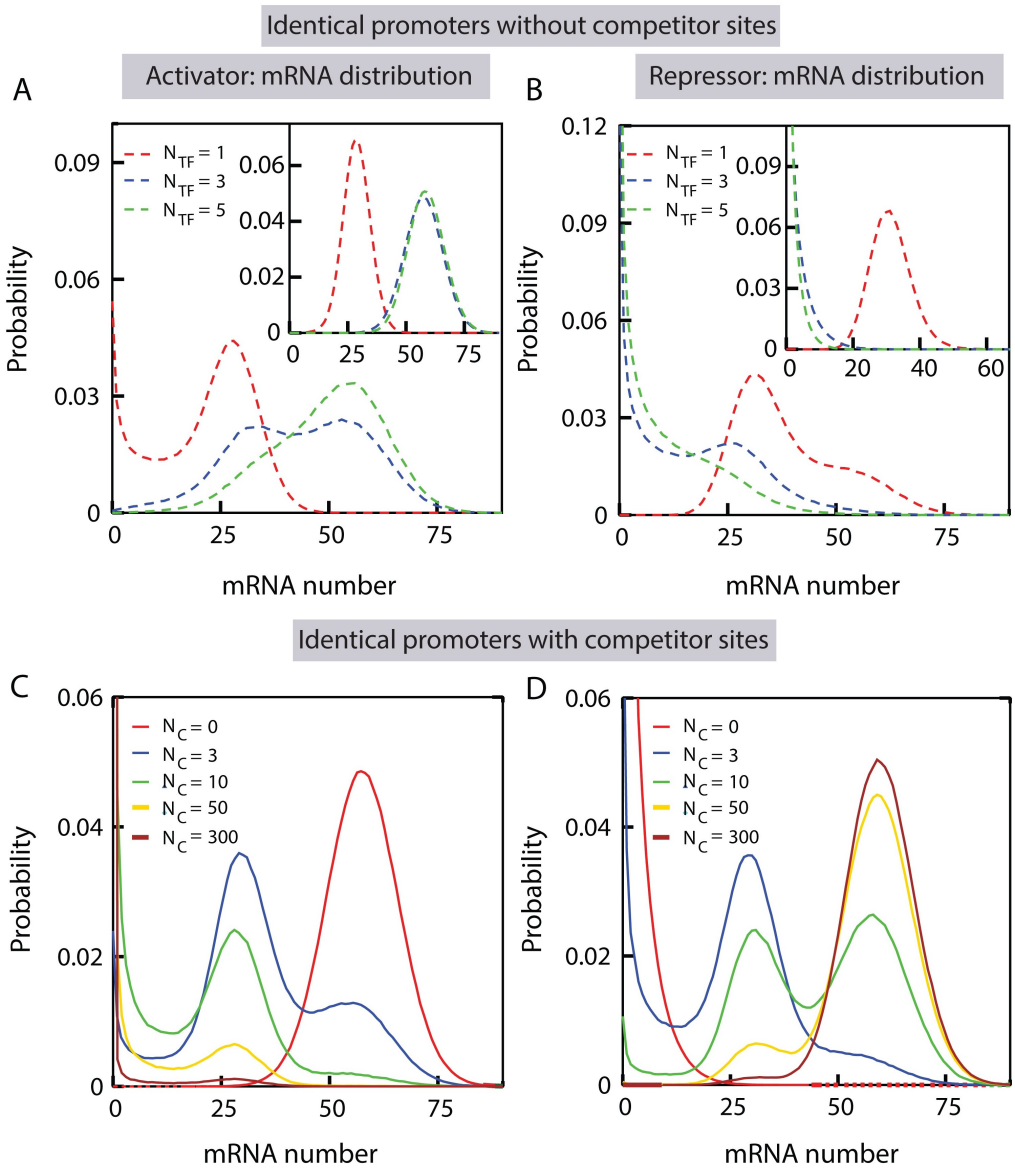
Introduction of competitor sites can lead to multi-modal mRNA distribution. (A-B) The steady-state number distributions of mRNA molecules produced by two identical promoters are shown in absence of any competitor sites, with varying number of activators (A) and repressors (B). The kinetic rates are taken from Table 1. Insets: The mRNA distributions with a faster TF binding rate (*k*_on_ = 0.027*s*^-1^) than the mRNA degradation rate (*γ* = 0.011*s*^−1^). Other parameters are as specified in Table 1. (C-D) The steady-state mRNA distributions for two identical promoters are shown in presence of competitor sites, when the TFs either act as activators (C) or repressors (D). For these plots, we choose a faster TF binding rate than mRNA degradation rate (as in the insets of A, B) such that the distributions are unimodal in absence of any competitor sites. The number of competitor sites is increased systematically keeping a fixed number of TFs (*N*_TF_ = 3). For simplicity we assume that the TF binding and unbinding rates to the promoters are the same as to the competitor sites.

The observations in the last two sections suggest that the mRNA distribution passes through an interesting regime when the TF number is comparable to the number of binding sites (promoter copies and competitor sites). In order to examine the features of mRNA distribution, we consider activator sharing between multiple promoter copies driving the expression of the same target gene. We find that multimodal mRNA distributions arise as activator number is tuned for a given number of promoter copies (see Fig 5A), when the binding and unbinding rates of activators (*k*_on_ and *k*_off_ respectively) to the promoters are much slower compared to the mRNA production and degradation rates (*r* and *γ* respectively)(see Table 1). Multi-modal mRNA distribution implies that mRNA molecules are present in multiple distinct abundances across an isogenic cell population. The appearance of multimodality at the mRNA level can be intuited by examining the steady state activator-promoter complex number distribution, since mRNA production is directly proportional to the number of activator-promoter complexes. In order to elucidate this point further, we consider the scenario when there are three activator copies, shared by two promoters. As evident, we can have three possible values of the complex number: zero, one or two. Since the binding and unbinding of the activator molecule to the promoters are much slower than the mRNA production and degradation rates, the promoters remain in activator bound and unbound states for a long time, such that sampling of mRNA molecules across a population can capture the effect of distinct relative abundances. Consequently in a genetically identical cell population, we may find three sub-populations, with one population having one complex producing ∼*r*/*γ* amount of mRNA molecules on average, another population having two complexes producing ∼2*r*/*γ* amount of mRNA molecules on average; and the other population having zero mRNA molecules on average due to the absence of any activator-promoter complexes. However, for our particular choice of parameters, the relative probability of having zero complexes becomes much smaller than having one or two complexes. Consequently the peak at zero vanishes, and we obtain a bi-modal mRNA distribution with two peaks, approximately at 2*r*/*γ* and *r*/*γ* (see the blue curve in Fig 5A). Nevertheless, with suitable choices of parameters one could recover all the three modes (see Fig J in S1 Text). In the limit of activator copy number being much greater than the promoter copy number, all the promoters are essentially occupied and the mRNA distribution approaches a uni-modal distribution, sharply peaked around *N*_P_*r*/*γ* (*N*_P_ being the number of promoter copies). For identical promoter copies competing for repressor molecules, we again find bi-modal mRNA distribution in certain parameter regime, as shown in Fig 5B.

The appearance of bimodal distribution is known theoretically for a single ‘two-state’ promoter switching between ‘on’ and ‘off’ states, when the state switching rates are slower than mRNA production and degradation rates [37, 91]. The fact that we consider more than one competing identical promoters leads to the possibility of obtaining more than two modes as we tune the TF copy number, since it allows for the formation of more than one TF-promoter complex. However, it is to be noted that multimodality can be observed only for a certain parameter regime, namely when the binding and unbinding rates of the TFs are much slower than mRNA production and degradation rates ({*r*, *γ*} > {*k*_on_, *k*_off_}). When this stringent condition is met, sharing of TFs among promoters alters the relative proportion of the different modes, making them either more or less prominent. On the other hand, if the condition is not met, the distribution becomes unimodal. For example, see the inset of Fig 5A and 5B, where we choose the TF binding rate *k*_on_ to be higher than the mRNA degradation rate *γ*.

Next, we investigate how the presence of competitor sites affects the distribution of mRNAs produced by multiple copies of a promoter of interest. In the case of activator sharing, one of the key findings is that the introduction of competitor sites can lead to multi-modality even when the binding rate of TFs to the promoter is faster than the degradation rate of the mRNA. In order to explain this further let us consider the example discussed above, where two competing promoters of interest produce a unimodal mRNA distribution in the absence of any competitor sites (Fig 5A, inset). By systematically increasing the number of competitor sites, we find that the unimodal mRNA distribution transforms into a multimodal distribution (see Fig 5C). This is because the activators can now also bind to the competitor sites, restricting the pool of freely available activators (ones that are not bound to any binding sites). Since the free activator number is restricted due to competition, the promoters can stay in unoccupied states for sufficiently long time, even when the binding rate of the activators are faster than the mRNA degradation rate. Hence the only requirement for observing multimodality is that the unbinding rate of activators to the promoter has to be slower than mRNA degradation rate, such that promoters can also stay occupied for a sufficiently long time, allowing the system to again sample the relative abundances of mRNA molecules. It should be noted that for quite a few known TFs, such as LacI, or lambda CI repressors in *E. Coli*, the unbinding rate from the promoter is slower or comparable to the mRNA degradation rate [42, 92]. This is also true for many TFs in yeast [93–95]. Please see the Methods section for a comprehensive discussion of the rates we choose.

Similarly, for two promoter copies sharing repressors with a number of competitor sites, our model predicts emergence of multi-modal mRNA distribution, as shown in Fig 5D. Just like the case of activators, different modes of mRNA distribution arise and diminish as the number of competitor sites is tuned. In the results above, we assumed for simplicity that the TFs have the same binding and unbinding rate to both the promoters and competitor sites. Nevertheless, our conclusions remain intact even when the TFs bind or unbind to the competitor sites with a different rate than to the promoters (see Fig K in S1 Text).

To further validate our model prediction that multi-modal mRNA distribution stems from the promoters of interest competing with the competitor sites for a pool of TFs, we also look at the Pearson correlation coefficient between the numbers of mRNA molecules produced from two promoters of interest. If the number of competitor sites is much smaller than the promoter number, and the TF number is much higher than the promoter number, effectively each promoter is expected to express independently. Thus, a nonzero correlation coefficient can be regarded as a signature of competition. For our choice of parameters, we see that the correlation starts from zero, reaches a minimum and then asymptotically approaches zero as the number of competitor sites is increased (See Fig L in S1 Text). Hence the rise of multimodality at the mRNA level coincides with nonzero values of correlation coefficient, signifying that this effect stems from the competition between the promoters and competitor sites. One central finding of this section is that altering the number of competitor sites and changing the number of TFs in the absence of competitor sites yield qualitatively distinct responses at the level of the mRNA distribution and correlation (see Fig M in S1 Text).

## Discussion

The impact of transcriptional dynamics on gene expression noise has been a topic of intense enquiry, in the field of quantitative regulatory biology [33–37, 39]. Most of these studies have investigated models of transcription, hinging on the properties of the cis regulatory elements, such as the number of TF binding sites, their binding strength, etc [39, 49, 54]. The key assumption these studies make is that we can treat each gene in isolation while dissecting their transcriptional dynamics and its impact on noise in expression. In reality this assumption often breaks down when a TF for a gene of interest gets shared by other genes or competitor sites on the chromosomal DNA or plasmids [55–61]. Hence in order to understand how transcriptional dynamics influences noise in gene expression, theoretical studies need to include the global effect of TF sharing.

The model of transcription developed here offers a way to incorporate the interplay of TF copy number and its binding sites and make predictions for how this interplay impacts gene expression noise. Predictions of this model, for the mean mRNA expression as a function of TF copy number is in agreement with recent bulk studies [67] (see Fig C in S1 Text). Moreover, for identical promoters competing for a pool of TFs, we find that both the variance and Fano factor of the mRNA distribution are non-monotonic functions of the TF copy number and show a peak when the TF and promoter copy numbers are comparable.

The incorporation of competitor sites into this model gives rise to intriguing predictions at the noise level. As has been expounded in recent studies [65], at the mean level, gene expression profiles from a wide range of competition scenarios in the presence of competitor sites can be collapsed onto a single master curve by considering the natural variable of the problem. However, at the level of variance this elegant universality ceases to exist. Although there exists a master curve in the variance as a function of the mean for a single promoter copy competing with multiple competitor sites, for more than one promoter copy, this result no longer holds. In other words, for more than one promoter copy, the simple picture of each promoter seeing an effective pool of TFs fails. Although introduction of competitor sites would imply a change in free TF concentration, one key implication of our results is that altering the number of competitor sites has a qualitatively different impact than directly changing the number of TFs in the absence of competitor sites.

As is evident, one major outcome of this study is that different scenarios of TF sharing lead to qualitatively distinct noise characteristics at the mRNA level. In a recent set of experiments in *E.coli*, Jones et al. [44] systematically tuned the number of LacI repressors and counted mRNA molecules across an isogenic population in order to demonstrate the effect of promoter architecture on gene expression noise. Similar strategies could be adopted to systematically test the different scenarios of TF resource sharing by altering the number and strength of competing binding sites, as we have outlined above.

We also find that the presence of competitor sites can further lead to a multi-modal distribution, so long as unbinding rates of TFs to the promoters of interest are slower than the mRNA production and degradation rates. Multi-modal distributions in expression, across an isogenic population can provide a fitness advantage in a changing environment, by giving rise to phenotypic diversity [96]. Certain cis regulatory elements have been demonstrated to lead to bimodal expression patterns [71, 91, 97, 98]. Here we demonstrate that a generic feature of promoters competing for a finite TF resource in the presence of competitor sites lead to a multi-modal distribution at the mRNA level. Whether such differential control is used by cells to control the shape and modality of the mRNA distributions is a fascinating question.

In general, the impact of global properties of transcription, such as the limited availability of TF [99] or sigma factor [100, 101] resources on gene expression noise remain poorly understood. In this light our theoretical framework provides a way of deciphering the global effect of genetic resource sharing on expression noise.

## Materials and methods

### Parameter selection

For *E.Coli*, there are several experimental studies that measure the degradation rates of the different mRNAs. In particular genome wide studies in *E.Coli* have shown that the average lifetime of mRNAs in E.coli is 2.5 minutes in the exponential phase and 4.5 minutes in the stationary phase respectively, as shown in Figure 3A of [95]. On the other hand numerous studies have measured the binding and unbinding rates of TFs to the promoters, as we have cited in the manuscript. For a lot of these TF such as the well-known Lac and Lambda cI repressors, the average residence times of minutes up to tens of minutes [42, 92, 102]. In other words the residence time is longer or comparable than the degradation rates of the mRNA molecules, justifying the assumption we make.

For eukaryotes, genome wide studies (see Figure 2A of [93]) in yeast have measured the lifetime of mRNA molecules from 4687 genes. These studies found that around 1700 genes have an average lifetime of less than 10 minutes. Although we do not know of genome wide studies that characterize the unbinding rates of all TFs to all binding sequences, we can find examples where the residence time of different TFs to the DNA were measured or inferred [94]. For TFs such as Rap I and Gcr I, the residence times are of the order or 10 minutes. As binding and unbinding rates of different TFs are discovered more of them might have a long residence time at the promoter.

### Computational tools

In order to compute the mRNA distribution across an isogenic population, we performed Gillespie simulations [85] using codes written in C++.

## Supporting information

S1 Text A single pdf file containing analytical calculations, discussion of activators with a basal rate of expression, and 13 supporting figures. (PDF)

## References

[1] Alberts B, Johnson A, Lewis J, Raff M, Roberts K, Walter P. Molecular Biology of the Cell. 4th ed New York: Garland Science; 2002.

[2] Zhang Y, McEwen AE, Crothers DM, Levene SD. Statistical-mechanical theory of DNA looping. Biophys J. 2006;90(6):1903–12. 10.1529/biophysj.105.070490 16361335 PMC1386771

[3] Phillips R, Kondev J, Theriot J, Garcia HG. Physical Biology of the Cell. 2nd ed New York: Garland Science; 2013.

[4] Cournac A, Plumbridge J. DNA looping in prokaryotes: experimental and theoretical approaches. J Bacteriol. 2013;195(6):1109–19. 10.1128/JB.02038-12 23292776 PMC3591992

[5] McAdams HH, Arkin A. It’s a noisy business! Genetic regulation at the nanomolar scale. Trends in genetics. 1999;15(2):65–9. 10.1016/S0168-9525(98)01659-X 10098409

[6] Rao CV, Wolf DM, Arkin AP. Control, exploitation and tolerance of intracellular noise. Nature. 2002;420(6912):231–237. 10.1038/nature01258 12432408

[7] Raser JM, O’Shea EK. Noise in gene expression: origins, consequences, and control. Science. 2005;309(5743):2010–3. 10.1126/science.1105891 16179466 PMC1360161

[8] Munsky B, Neuert G, van Oudenaarden A. Using gene expression noise to understand gene regulation. Science. 2012;336(6078):183–7. 10.1126/science.1216379 22499939 PMC3358231

[9] Raj A, van Oudenaarden A. Nature, nurture, or chance: stochastic gene expression and its consequences. Cell. 2008;135(2):216–26. 10.1016/j.cell.2008.09.050 18957198 PMC3118044

[10] Elowitz MB, Levine AJ, Siggia ED, Swain PS. Stochastic gene expression in a single cell. Science. 2002;297(5584):1183–6. 10.1126/science.1070919 12183631

[11] Sanchez A, Golding I. Genetic determinants and cellular constraints in noisy gene expression. Science. 2013;342(6163):1188–93. 10.1126/science.1242975 24311680 PMC4045091

[12] Fraser HB, Hirsh AE, Giaever G, Kumm J, Eisen MB. Noise minimization in eukaryotic gene expression. PLoS biology. 2004;2(6):e137 10.1371/journal.pbio.0020137 15124029 PMC400249

[13] Bai L, Charvin G, Siggia ED, Cross FR. Nucleosome-depleted regions in cell-cycle-regulated promoters ensure reliable gene expression in every cell cycle. Developmental cell. 2010;18(4):544–55. 10.1016/j.devcel.2010.02.007 20412770 PMC2867244

[14] Newman JRS, Ghaemmaghami S, Ihmels J, Breslow DK, Noble M, DeRisi JL, et al Single-cell proteomic analysis of S. cerevisiae reveals the architecture of biological noise. Nature. 2006;441(7095):840–6. 10.1038/nature04785 16699522

[15] Arias AM, Hayward P. Filtering transcriptional noise during development: concepts and mechanisms. Nat Rev Genet. 2006;7:34–44. 10.1038/nrg1750 16369570

[16] Kussell E, Leibler S. Phenotypic diversity, population growth, and information in fluctuating environments. Science. 2005;309(5743):2075–8. 10.1126/science.1114383 16123265

[17] Weinberger LS, Burnett JC, Toettcher JE, Arkin AP, Schaffer DV. Stochastic gene expression in a lentiviral positive-feedback loop: HIV-1 Tat fluctuations drive phenotypic diversity. Cell. 2005;122(2):169–82. 10.1016/j.cell.2005.06.006 16051143

[18] Kaern M, Elston TC, Blake WJ, Collins JJ. Stochasticity in gene expression: from theories to phenotypes. Nat Rev Genet. 2005;. 10.1038/nrg1615 15883588

[19] Blake WJ, Balazsi G, Kohanski MA, Isaacs FJ, Murphy KF, Kuang Y, et al Phenotypic consequences of promoter-mediated transcriptional noise. Mol Cell. 2006;24(6):853–65. 10.1016/j.molcel.2006.11.003 17189188

[20] Thattai M, van Oudenaarden A. Stochastic Gene Expression in Fluctuating Environments. Genetics. 2004;167(1):523–530. 10.1534/genetics.167.1.523 15166174 PMC1470854

[21] Losick R, Desplan C. Stochasticity and cell fate. Science. 2008;320(5872):65–8. 10.1126/science.1147888 18388284 PMC2605794

[22] López-Maury L, Marguerat S, Bähler J. Tuning gene expression to changing environments: from rapid responses to evolutionary adaptation. Nat Rev Genet. 2008;9(8):583–93. 10.1038/nrg2398 18591982

[23] Rotem E, Loinger A, Ronin I, Levin-Reisman I, Gabay C, Shoresh N, et al Regulation of phenotypic variability by a threshold-based mechanism underlies bacterial persistence. Proc Natl Acad Sci U S A. 2010;107(28):12541–6. 10.1073/pnas.1004333107 20616060 PMC2906590

[24] Balázsi G, van Oudenaarden A, Collins JJ. Cellular decision making and biological noise: from microbes to mammals. Cell. 2011;144(6):910–25. 10.1016/j.cell.2011.01.030 21414483 PMC3068611

[25] Rister J, Desplan C, Vasiliauskas D. Establishing and maintaining gene expression patterns: insights from sensory receptor patterning. Development. 2013;140(3):493–503. 10.1242/dev.079095 23293281 PMC3561783

[26] Charlebois DA, Abdennur N, Kaern M. Gene Expression Noise Facilitates Adaptation and Drug Resistance Independently of Mutation. Phys Rev Lett. 2011;107:218101 10.1103/PhysRevLett.107.218101 22181928

[27] Brock A, Chang H, Huang S. Non-genetic heterogeneity–a mutation-independent driving force for the somatic evolution of tumours. Nat Rev Genet. 2009;10(5):336–42. 10.1038/nrg2556 19337290

[28] Sharma SV, Lee DY, Li B, Quinlan MP, Takahashi F, Maheswaran S, et al A chromatin-mediated reversible drug-tolerant state in cancer cell subpopulations. Cell. 2010;141(1):69–80. 10.1016/j.cell.2010.02.027 20371346 PMC2851638

[29] Eldar A, Elowitz MB. Functional roles for noise in genetic circuits. Nature. 2010;467(7312):167–73. 10.1038/nature09326 20829787 PMC4100692

[30] Tsimring LS. Noise in biology. Rep Prog Phys. 2014;77(2):026601 10.1088/0034-4885/77/2/026601 24444693 PMC4033672

[31] Chalancon G, Ravarani CNJ, Balaji S, Martinez-Arias A, Aravind L, Jothi R, et al Interplay between gene expression noise and regulatory network architecture. Trends in Genetics. 2012;28(5):221–232. 10.1016/j.tig.2012.01.006 22365642 PMC3340541

[32] Sanchez A, Choubey S, Kondev J. Regulation of noise in gene expression. Annu Rev Biophys. 2013;42:469–91. 10.1146/annurev-biophys-083012-130401 23527780

[33] Sanchez A, Kondev J. Transcriptional control of noise in gene expression. Proc Natl Acad Sci U S A. 2008;105(13):5081–6. 10.1073/pnas.0707904105 18353986 PMC2278180

[34] Elgart V, Jia T, Fenley AT, Kulkarni R. Connecting protein and mRNA burst distributions for stochastic models of gene expression. Phys Biol. 2011;8(4):046001 10.1088/1478-3975/8/4/046001 21490380

[35] Singh A, Weinberger LS. Stochastic gene expression as a molecular switch for viral latency. Curr Opin Microbiol. 2009;12(4):460–6. 10.1016/j.mib.2009.06.016 19595626 PMC2760832

[36] Kumar N, Singh A, Kulkarni RV. Transcriptional Bursting in Gene Expression: Analytical Results for General Stochastic Models. PLoS Comput Biol. 2015;11(10):e1004292 10.1371/journal.pcbi.1004292 26474290 PMC4608583

[37] Sanchez A, Choubey S, Kondev J. Stochastic models of transcription: From single molecules to single cells. Methods. 2013;62(1):13—25. doi:10.1016/j.ymeth.2013.03.026. 23557991

[38] Kumar N, Platini T, Kulkarni RV. Exact Distributions for Stochastic Gene Expression Models with Bursting and Feedback. Phys Rev Lett. 2014;113:268105 10.1103/PhysRevLett.113.268105 25615392

[39] Choubey S, Kondev J, Sanchez A. Deciphering Transcriptional Dynamics In Vivo by Counting Nascent RNA Molecules. PLoS Comput Biol. 2015;11(11):e1004345 10.1371/journal.pcbi.1004345 26544860 PMC4636183

[40] Lipniacki T, Paszek P, Marciniak-Czochra A, Brasier AR, Kimmel M. Transcriptional stochasticity in gene expression. J Theor Biol. 2006;238(2):348—367. doi:10.1016/j.jtbi.2005.05.032. 16039671

[41] Yu J, Xiao J, Ren X, Lao K, Xie XS. Probing gene expression in live cells, one protein molecule at a time. Science. 2006;311(5767):1600–3. 10.1126/science.1119623 16543458

[42] Elf J, Li GW, Xie XS. Probing transcription factor dynamics at the single-molecule level in a living cell. Science. 2007;316(5828):1191–4. 10.1126/science.1141967 17525339 PMC2853898

[43] Choi PJ, Cai L, Frieda K, Xie XS. A stochastic single-molecule event triggers phenotype switching of a bacterial cell. Science. 2008;322(5900):442–6. 10.1126/science.1161427 18927393 PMC2819113

[44] Jones DL, Brewster RC, Phillips R. Promoter architecture dictates cell-to-cell variability in gene expression. Science. 2014;346(6216):1533–6. 10.1126/science.1255301 25525251 PMC4388425

[45] Sharon E, van Dijk D, Kalma Y, Keren L, Manor O, Yakhini Z, et al Probing the effect of promoters on noise in gene expression using thousands of designed sequences. Genome research. 2014;24(10):1698–706. 10.1101/gr.168773.113 25030889 PMC4199362

[46] Raj A, Peskin CS, Tranchina D, Vargas DY, Tyagi S. Stochastic mRNA synthesis in mammalian cells. PLoS Biol. 2006;4(10):e309 10.1371/journal.pbio.0040309 17048983 PMC1563489

[47] Chubb JR, Trcek T, Shenoy SM, Singer RH. Transcriptional pulsing of a developmental gene. Curr Biol. 2006;16(10):1018–25. 10.1016/j.cub.2006.03.092 16713960 PMC4764056

[48] Gandhi SJ, Zenklusen D, Lionnet T, Singer RH. Transcription of functionally related constitutive genes is not coordinated. Nat Struct Mol Biol. 2011;18(1):27–34. 10.1038/nsmb.1934 21131977 PMC3058351

[49] Vilar JM, Leibler S. DNA looping and physical constraints on transcription regulation. J Mol Biol. 2003;331(5):981–9. 10.1016/S0022-2836(03)00764-2 12927535

[50] Bilu Y, Barkai N. The design of transcription-factor binding sites is affected by combinatorial regulation. Genome Biology. 2005;6(12):1–10. 10.1186/gb-2005-6-12-r103 16356266 PMC1414079

[51] Shahrezaei V, Swain PS. Analytical distributions for stochastic gene expression. Proc Natl Acad Sci U S A. 2008;105(45):17256–61. 10.1073/pnas.0803850105 18988743 PMC2582303

[52] Segal E, Widom J. From DNA sequence to transcriptional behaviour: a quantitative approach. Nat Rev Genet. 2009;10(7):443–56. 10.1038/nrg2591 19506578 PMC2719885

[53] Gertz J, Siggia ED, Cohen BA. Analysis of combinatorial cis-regulation in synthetic and genomic promoters. Nature. 2009;457(7226):215–8. 10.1038/nature07521 19029883 PMC2677908

[54] Sanchez A, Garcia HG, Jones D, Phillips R, Kondev J. Effect of promoter architecture on the cell-to-cell variability in gene expression. PLoS Comput Biol. 2011;7(3):e1001100 10.1371/journal.pcbi.1001100 21390269 PMC3048382

[55] Luria SE, Dulbecco R. Genetic Recombinations Leading to Production of Active Bacteriophage from Ultraviolet Inactivated Bacteriophage Particles. Genetics. 1949;34(2):93–125. 17247312 10.1093/genetics/34.2.93 PMC1209443

[56] Guido NJ, Wang X, Adalsteinsson D, McMillen D, Hasty J, Cantor CR, et al A bottom-up approach to gene regulation. Nature. 2006;439(7078):856–60. 10.1038/nature04473 16482159

[57] Bremer H, Dennis PP. Modulation of Chemical Composition and Other Parameters of the Cell by Growth Rate In: e al NF, editor. In *Escherichia coli* and Salmonella Cellular and Molecular Biology. Washington DC: ASM Press; 1996 p. 1553–1569.

[58] Wang RL, Stec A, Hey J, Lukens L, Doebley J. The limits of selection during maize domestication. Nature. 1999;398(6724):236–9. 10.1038/18435 10094045

[59] Navarro-Quezada A, Schoen DJ. Sequence evolution and copy number of Ty1-copia retrotransposons in diverse plant genomes. Proc Natl Acad Sci U S A. 2002;99(1):268–73. 10.1073/pnas.012422299 11752395 PMC117550

[60] Aitman TJ, Dong R, Vyse TJ, Norsworthy PJ, Johnson MD, Smith J, et al Copy number polymorphism in Fcgr3 predisposes to glomerulonephritis in rats and humans. Nature. 2006;439(7078):851–5. 10.1038/nature04489 16482158

[61] Hanada T, Hashizume Y, Tokuhara N, Takenaka O, Kohmura N, Ogasawara A, et al Perampanel: a novel, orally active, noncompetitive AMPA-receptor antagonist that reduces seizure activity in rodent models of epilepsy. Epilepsia. 2011;52(7):1331–40. 10.1111/j.1528-1167.2011.03109.x 21635236

[62] Gama-Castro S, Jimenez-Jacinto V, Peralta-Gil M, Santos-Zavaleta A, Penaloza-Spinola MI, Contreras-Moreira B, et al RegulonDB (version 6.0): gene regulation model of Escherichia coli K-12 beyond transcription, active (experimental) annotated promoters and Textpresso navigation. Nucleic Acids Res. 2008;36:D120–4. 10.1093/nar/gkm994 18158297 PMC2238961

[63] Shimada T, Kawai T, Takeda K, Matsumoto M, Inoue J, Tatsumi Y, et al IKK-i, a novel lipopolysaccharide-inducible kinase that is related to IkappaB kinases. International immunology. 1999;11(8):1357–62. 10.1093/intimm/11.8.1357 10421793

[64] Brewster RC, Jones DL, Phillips R. Tuning promoter strength through RNA polymerase binding site design in *Escherichia coli*. PLoS Comput Biol. 2012;8(12):e1002811 10.1371/journal.pcbi.1002811 23271961 PMC3521663

[65] Weinert FM, Brewster RC, Rydenfelt M, Phillips R, Kegel WK. Scaling of gene expression with transcription-factor fugacity. Phys Rev Lett. 2014;113(25):1–5. 10.1103/PhysRevLett.113.258101 PMC4386862 25554908

[66] Lovely GA, Brewster RC, Schatz DG, Baltimore D, Phillips R. Single-molecule analysis of RAG-mediated V(D)J DNA cleavage. Proc Natl Acad Sci U S A. 2015;112(14):E1715–23. 10.1073/pnas.1503477112 25831509 PMC4394307

[67] Brewster RC, Weinert FM, Garcia HG, Song D, Rydenfelt M, Phillips R. The transcription factor titration effect dictates level of gene expression. Cell. 2014;156(6):1312–23. 10.1016/j.cell.2014.02.022 24612990 PMC4080642

[68] Burger A, Walczak AM, Wolynes PG. Abduction and asylum in the lives of transcription factors. Proc Natl Acad Sci U S A. 2010;107(9):4016–21. 10.1073/pnas.0915138107 20160109 PMC2840107

[69] Burger A, Walczak AM, Wolynes PG. Influence of decoys on the noise and dynamics of gene expression. Phys Rev E Stat Nonlin Soft Matter Phys. 2012;86(4 Pt 1):041920 10.1103/PhysRevE.86.041920 23214628

[70] Soltani M, Bokes P, Fox Z, Singh A. Nonspecific transcription factor binding can reduce noise in the expression of downstream proteins. Physical Biology. 2015;12(5):055002 10.1088/1478-3975/12/5/055002 26267711

[71] Bokes P, Singh A. Protein copy number distributions for a self-regulating gene in the presence of decoy binding sites. PloS one. 2015;10(3):e0120555 10.1371/journal.pone.0120555 25811868 PMC4374843

[72] Karapetyan S, Buchler NE. Role of DNA binding sites and slow unbinding kinetics in titration-based oscillators. Phys Rev E. 2015;92:062712 10.1103/PhysRevE.92.062712 PMC4777296 26764732

[73] Rydenfelt M, Cox RS, Garcia H, Phillips R. Statistical mechanical model of coupled transcription from multiple promoters due to transcription factor titration. Phys Rev E. 2014;89:012702 10.1103/PhysRevE.89.012702 PMC4043999 24580252

[74] Rydenfelt M, Garcia HG, Cox R, Phillips R. The Influence of Promoter Architectures and Regulatory Motifs on Gene Expression in *Escherichia coli*. PLoS ONE. 2014;9(12):e114347 10.1371/journal.pone.0114347 25549361 PMC4280137

[75] Kepler TB, Elston TC. Stochasticity in transcriptional regulation: origins, consequences, and mathematical representations. Biophys J. 2001;81:3116–36. 10.1016/S0006-3495(01)75949-8 11720979 PMC1301773

[76] Karmakar R, Bose I. Graded and binary responses in stochastic gene expression. Physical biology. 2004;1:197–204. 10.1088/1478-3967/1/4/001 16204839

[77] Paulsson J. Models of stochastic gene expression. Phys Life Rev. 2005;2(2):157–175. 10.1016/j.plrev.2005.03.003

[78] Dattani J, Barahona M. Stochastic models of gene transcription with upstream drives: exact solution and sample path characterization. arXiv:160507124. 2016; p. 19. 10.1098/rsif.2016.0833 PMC5310734 28053113

[79] Garcia HG, Phillips R. Quantitative dissection of the simple repression input-output function. Proc Natl Acad Sci U S A. 2011;108(29):12173–8. 10.1073/pnas.1015616108 21730194 PMC3141941

[80] Firman T, Ghosh K. Competition enhances stochasticity in biochemical reactions. J Chem Phys. 2013;139(12). doi:10.1063/1.4816527. 24089727

[81] Peccoud J, Ycart B. Markovian modeling of gene product synthesis. Theor Popul Biol. 1995;48:222–234. 10.1006/tpbi.1995.1027

[82] Sasai M, Wolynes PG. Stochastic gene expression as a many-body problem. Proc Natl Acad Sci U S A. 2003;100(5):2374–2379. 10.1073/pnas.2627987100 12606710 PMC151348

[83] Golding I, Paulsson J, Zawilski SM, Cox EC. Real-time kinetics of gene activity in individual bacteria. Cell. 2005;123(6):1025–36. 10.1016/j.cell.2005.09.031 16360033

[84] Cai L, Friedman N, Xie XS. Stochastic protein expression in individual cells at the single molecule level. Nature. 2006;440(7082):358–62. 10.1038/nature04599 16541077

[85] Gillespie DT. General Method for Numerically Simulating Stochastic Time Evolution of Coupled Chemical-Reactions. J Comput Phys. 1976;22(4):403–434. 10.1016/0021-9991(76)90041-3

[86] Wong OK, Guthold M, Erie DA, Gelles J. Interconvertible lac repressor-DNA loops revealed by single-molecule experiments. PLoS Biol. 2008;6(9):e232 10.1371/journal.pbio.0060232 18828671 PMC2553838

[87] Kennell D, Riezman H. Transcription and translation initiation frequencies of the *Escherichia coli lac* operon. J Mol Biol. 1977;114(1):1–21. 10.1016/0022-2836(77)90279-0 409848

[88] Bakk A, Metzler R, Sneppen K. Sensitivity of OR in phage lambda. Biophys J. 2004;86(1 Pt 1):58–66. 10.1016/S0006-3495(04)74083-7 14695249 PMC1303827

[89] Wunderlich Z, Depace AH. Modeling transcriptional networks in Drosophila development at multiple scales. Curr Opin Genet Dev. 2011;. 10.1016/j.gde.2011.07.005 21889888

[90] Lee TH, Maheshri N. A regulatory role for repeated decoy transcription factor binding sites in target gene expression. Mol Syst Biol. 2012;8:576 10.1038/msb.2012.7 22453733 PMC3322331

[91] Iyer-Biswas S, Hayot F, Jayaprakash C. Stochasticity of gene products from transcriptional pulsing. Phys Rev E Stat Nonlin Soft Matter Phys. 2009;79(3 Pt 1):031911 10.1103/PhysRevE.79.031911 19391975

[92] Dodd IB, Shearwin KE, Perkins AJ, Burr T, Hochschild A, Egan JB. Cooperativity in long-range gene regulation by the lambda CI repressor. Genes Dev. 2004;18(3):344–54. 10.1101/gad.1167904 14871931 PMC338286

[93] Wang Y, Liu CL, Storey JD, Tibshirani RJ, Herschlag D, Brown PO. Precision and functional specificity in mRNA decay. Proc Natl Acad Sci U S A. 2002;99(9):5860–5865. 10.1073/pnas.092538799 11972065 PMC122867

[94] Carey LB, van Dijk D, Sloot PMA, Kaandorp JA, Segal E. Promoter Sequence Determines the Relationship between Expression Level and Noise. PLOS Biology. 2013;11(4):1–15. 10.1371/journal.pbio.1001528 PMC3614515 23565060

[95] Chen H, Shiroguchi K, Ge H, Xie XS. Genome-wide study of mRNA degradation and transcript elongation in Escherichia coli. Molecular Systems Biology. 2015;11(1). 10.15252/msb.20145794 PMC4332155 25583150

[96] Acar M, Mettetal JT, van Oudenaarden A. Stochastic switching as a survival strategy in fluctuating environments. Nat Genet. 2008;40(4):471–5. 10.1038/ng.110 18362885

[97] To TL, Maheshri N. Noise can induce bimodality in positive transcriptional feedback loops without bistability. Science (New York, NY). 2010;327(5969):1142–5. 10.1126/science.1178962 20185727

[98] Ochab-Marcinek A, Tabaka M. Bimodal gene expression in noncooperative regulatory systems. Proc Natl Acad Sci U S A. 2010;107(51):22096–101. 10.1073/pnas.1008965107 21135209 PMC3009792

[99] Lorenzin F, Benary U, Baluapuri A, Walz S, Jung LA, von Eyss B, et al Different promoter affinities account for specificity in MYC-dependent gene regulation. eLife. 2016;5:e15161 10.7554/eLife.15161 27460974 PMC4963202

[100] Mauri M, Klumpp S. A Model for Sigma Factor Competition in Bacterial Cells. PLoS Comput Biol. 2014;10(10):e1003845 10.1371/journal.pcbi.1003845 25299042 PMC4191881

[101] Locke JCW, Young JW, Fontes M, Jiménez MJH, Elowitz MB. Stochastic Pulse Regulation in Bacterial Stress Response. Science. 2011;334(6054):366–369. 10.1126/science.1208144 21979936 PMC4100694

[102] Hammar P, Wallden M, Fange D, Persson F, Baltekin O, Ullman G, et al Direct measurement of transcription factor dissociation excludes a simple operator occupancy model for gene regulation. Nat Genet. 2014;46(4):405–408. 10.1038/ng.2905 24562187 PMC6193529

