# Supplementary material for: Effect of transcription factor resource sharing on gene expression noise
Source: PLoS Comput Biol. 2017 Apr 17;13(4):e1005491. doi: 10.1371/journal.pcbi.1005491 (PMC5411101; doi:10.1371/journal.pcbi.1005491)
Supplement: S1 Text — (PDF) [file pcbi.1005491.s001.pdf]

Supporting Information

Dipjyoti Das,[1,*] Supravat Dey,[2,*] Robert Brewster,[3,4,†] and Sandeep Choubey[5,6,†]

[1]*Department of Molecular, Cellular and Developmental Biology, Yale University, New Haven, CT 06511.*
[2]*Laboratoire Charles Coulomb, Université Montpellier and CNRS, 34095 Montpellier, France.*
[3]*Program in Systems Biology, University of Massachusetts Medical School 368 Plantation St., Worcester, MA 01605.*
[4]*Department of Microbiology and Physiological Systems, University of Massachusetts Medical School, 368 Plantation St., Worcester, MA 01605.*[‡]
[5]*FAS Center for Systems Biology, Harvard University, Cambridge, MA 02138.*
[6]*Department of Molecular and Cellular Biology, Harvard University, Cambridge, MA 02138.*[§]

(Dated: April 6, 2017)

A general model of many identical promoters competing for TFs

We develop a general model where multiple identical promoters compete for a pool of transcription factors (TFs). A TF binds to a promoter at a rate $k_{\rm on}$, and it unbinds from the promoter at a rate $k_{\rm off}$. In other words, the TF-promoter complexes form at a rate $k_{\rm on}$ per TF per promoter, while the dissociation rate per complex is $k_{\rm off}$. Each of these TF-promoter complexes produces an mRNA molecule at a rate $r_{\rm bound}$, while every 'TF-free' promoter produces an mRNA molecule at a rate $r_{\rm free}$. The mRNA molecules subsequently degrade at a rate γ. We consider $N_{\rm TF}$ number of TFs shared by $N_{\rm P}$ number of identical promoters. By employing a stochastic framework, we monitor the time evolution of two random variables: the instantaneous number of TF-promoter complexes, $n(t)$ and the instantaneous number of mRNA molecules, $m(t)$. All the above processes can be summarized by the following five reactions:

$$
\begin{aligned}
R_1 &: \quad n \xrightarrow{k_{\rm on}(N_{\rm TF}-n)(N_{\rm P}-n)} n+1 \quad \text{(Binding of a TF to a promoter)} \\
R_2 &: \quad n \xrightarrow{k_{\rm off}n} n-1 \quad \text{(Unbinding of a TF from a promoter)} \\
R_3 &: \quad m \xrightarrow{r_{\rm bound}n} m+1 \quad \text{(Production of mRNA from TF-bound promoters)} \\
R_4 &: \quad m \xrightarrow{r_{\rm free}(N_{\rm P}-n)} m+1 \quad \text{(Production of mRNA from TF-free promoters)} \\
R_5 &: \quad m \xrightarrow{\gamma m} m-1 \quad \text{(Decay of mRNA).}
\end{aligned} \tag{1}
$$

Note that the bare rates $k_{\rm off}, r_{\rm bound}, r_{\rm free}$, and γ should be interpreted as the probabilities of occurring the respective reactions per unit time per molecule (reactions R_2, R_3, R_4, R_5 in Eq. 1). However, the binding rate of the TFs, $k_{\rm on}$ is the probability per unit time of forming a complex, per TF per promoter. Thus the effective instantaneous binding rate is proportional to the product of freely available TFs and promoters i.e. $(N_{\rm TF}-n)(N_{\rm P}-n)$ (reaction R_1 in Eq. 1). Also note that finite numbers of TFs and promoters limit the maximum possible number of complexes — this will be the minimum of the TF number and promoter number i.e. $max(n) = min(N_{\rm TF}, N_{\rm P})$.

Using the reactions defined in Eq. 1, we can write down the master equation, which is a time-evolution equation for the joint probability distribution $P(n, m, t)$ of having n complexes and m mRNA molecules at a time t. The master equation reads,

$$
\begin{aligned}
\frac{dP(n,m,t)}{dt} &= k_{\rm on}(N_{\rm TF}-n+1)(N_{\rm P}-n+1)P(n-1,m,t) + k_{\rm off}(n+1)P(n+1,m,t) + r_{\rm bound}nP(n,m-1,t) \\
&\quad + r_{\rm free}(N_{\rm P}-n)P(n,m-1,t) + \gamma(m+1)P(n,m+1,t) \\
&\quad - [k_{\rm on}(N_{\rm TF}-n)(N_{\rm P}-n) + k_{\rm off}n + r_{\rm bound}n + r_{\rm free}(N_{\rm P}-n) + \gamma m]P(n,m,t).
\end{aligned} \tag{2}
$$

The above equation is built by counting all the possible steps from the reactions (Eq. 1) that lead to either a change in n or in m.

---

[*] These two authors contributed equally to the work
[†] Corresponding authors
[‡] Electronic address: `Robert.Brewster@umassmed.edu`
[§] Electronic address: `schoubey@fas.harvard.edu`

The general model described above can be reduced to different special cases for when the TFs act either as activators or repressors. For example, by putting $r_{\text{bound}} = r$ and $r_{\text{free}} = 0$ into Eq. 2 we can obtain the master equation for the case of activators without any basal expression level — this actually leads to Eq. 1 described in the main text. If both r_{bound} and r_{free} are nonzero, the situation corresponds to the case of activators with a basal expression, where r_{free} is simply the basal rate of transcription. Meanwhile, the case of repressors can be obtained by using $r_{\text{bound}} = 0$ and $r_{\text{free}} = r$.

We now proceed to calculate all the relevant moments of the joint distribution, which can be defined as

$$\text{mean}: \quad \langle m \rangle = \sum_{n=0}^{min(N_{\text{TF}},N_{\text{P}})} \sum_{m=0}^{\infty} mP(n,m,t) \quad ; \quad \langle n \rangle = \sum_{n=0}^{min(N_{\text{TF}},N_{\text{P}})} \sum_{m=0}^{\infty} nP(n,m,t)$$

$$\text{2nd moment}: \quad \langle m^2 \rangle = \sum_{n=0}^{min(N_{\text{TF}},N_{\text{P}})} \sum_{m=0}^{\infty} m^2 P(n,m,t) \quad ; \quad \langle n^2 \rangle = \sum_{n=0}^{min(N_{\text{TF}},N_{\text{P}})} \sum_{m=0}^{\infty} n^2 P(n,m,t) \quad .... \text{ etc.}$$

In principle, we can obtain the time-evolution equations for the moments starting from the master equation (Eq. 2). For example, multiplying Eq. 2 by m and then summing over all possible values of n and m, one could get the equation for the mean mRNA copy number, $\langle m \rangle$ as below

$$\frac{d\langle m \rangle}{dt} = r_{\text{bound}}\langle n \rangle + r_{\text{free}}(N_{\text{P}} - \langle n \rangle) - \gamma \langle m \rangle \tag{3}$$

Proceeding in similar ways we further obtain

$$\frac{d\langle n \rangle}{dt} = k_{\text{on}}\langle (N_{\text{TF}} - n)(N_{\text{P}} - n) \rangle - k_{\text{off}}\langle n \rangle \tag{4}$$

$$\frac{d\langle m^2 \rangle}{dt} = 2r_{\text{bound}}\langle mn \rangle + r_{\text{bound}}\langle n \rangle + 2r_{\text{free}}(N_{\text{P}}\langle m \rangle - \langle mn \rangle) + r_{\text{free}}(N_{\text{P}} - \langle n \rangle) + \gamma \langle m \rangle - 2\gamma \langle m^2 \rangle \tag{5}$$

$$\frac{d\langle mn \rangle}{dt} = k_{\text{on}}\langle m(N_{\text{TF}} - n)(N_{\text{P}} - n) \rangle - k_{\text{off}}\langle mn \rangle + r_{\text{bound}}\langle n^2 \rangle + r_{\text{free}}(N_{\text{P}}\langle n \rangle - \langle n^2 \rangle) - \gamma \langle mn \rangle \tag{6}$$

$$\frac{d\langle n^2 \rangle}{dt} = 2k_{\text{on}}\langle n(N_{\text{TF}} - n)(N_{\text{P}} - n) \rangle + k_{\text{on}}\langle (N_{\text{TF}} - n)(N_{\text{P}} - n) \rangle - 2k_{\text{off}}\langle n^2 \rangle + k_{\text{off}}\langle n \rangle \tag{7}$$

Note that above equations do not form a closed set, which hinders the calculation of exact closed-form solutions. For example, equation for $\langle n \rangle$ contains $\langle n^2 \rangle$, while equation for $\langle n^2 \rangle$ contains $\langle n^3 \rangle$ and so on; this forms an infinite hierarchy. The hierarchy must be broken with appropriate approximations to obtain truncated equations. We therefore make the following approximations

$$\begin{aligned} \langle (n - \langle n \rangle)^3 \rangle \approx 0 \qquad & \text{or,} \quad \langle n^3 \rangle \approx 3\langle n^2 \rangle \langle n \rangle - 2\langle n \rangle^3 \\ \langle (m - \langle m \rangle)(n - \langle n \rangle)^2 \rangle \approx 0 \qquad & \text{or,} \quad \langle mn^2 \rangle \approx 2\langle mn \rangle \langle n \rangle - 2\langle m \rangle \langle n \rangle^2 + \langle m \rangle \langle n^2 \rangle \end{aligned} \tag{8}$$

In above approximations, the higher order moments beyond the second-order are assumed to be zero. Essentially, the distribution is assumed to be symmetric around the mean.

We focus in the steady-state limit ($t \to \infty$), where the distribution does not change with time. In other words, the time-derivatives in the left-hand sides of the Eqs 3-7 can be set to zero. Ultimately, with the above approximations (Eq. 8) the steady-state equations for moments become

$$r_{\text{bound}}\langle n \rangle + r_{\text{free}}(N_{\text{P}} - \langle n \rangle) - \gamma \langle m \rangle = 0 \tag{9}$$

$$k_{\text{on}}[N_{\text{TF}}N_{\text{P}} - (N_{\text{TF}} + N_{\text{P}})\langle n \rangle + \langle n^2 \rangle] - k_{\text{off}}\langle n \rangle = 0 \tag{10}$$

$$2r_{\text{bound}}\langle mn \rangle + r_{\text{bound}}\langle n \rangle + 2r_{\text{free}}(N_{\text{P}}\langle m \rangle - \langle mn \rangle) + r_{\text{free}}(N_{\text{P}} - \langle n \rangle) + \gamma \langle m \rangle - 2\gamma \langle m^2 \rangle = 0 \tag{11}$$

$$\begin{aligned} k_{\text{on}}[N_{\text{TF}}N_{\text{P}} - (N_{\text{TF}} + N_{\text{P}})\langle mn \rangle + (2\langle mn \rangle \langle n \rangle - 2\langle m \rangle \langle n \rangle^2 + \langle m \rangle \langle n^2 \rangle)] - k_{\text{off}}\langle mn \rangle + r_{\text{bound}}\langle n^2 \rangle & \\ + r_{\text{free}}(N_{\text{P}}\langle n \rangle - \langle n^2 \rangle) - \gamma \langle mn \rangle & \approx 0 \end{aligned} \tag{12}$$

$$\begin{aligned} 2k_{\text{on}}[N_{\text{TF}}N_{\text{P}} - (N_{\text{TF}} + N_{\text{P}})\langle n^2 \rangle + (3\langle n^2 \rangle \langle n \rangle - 2\langle n \rangle^3)] + k_{\text{on}}[N_{\text{TF}}N_{\text{P}} - (N_{\text{TF}} + N_{\text{P}})\langle n \rangle + \langle n^2 \rangle] & \\ - 2k_{\text{off}}\langle n^2 \rangle + k_{\text{off}}\langle n \rangle & \approx 0 \end{aligned} \tag{13}$$

Above coupled equations (Eqs 9-13) form a simple closed set, which can be solved numerically to obtain the steady-state means and variances. Note that these set of equations are quadratic in nature. Thus there would be three set of solutions. However, the only physically meaningful solution can be identified by noting two constraints: (i) $var(n) > 0$, $var(m) > 0$; and (ii) $\langle n \rangle \leq min(N_{\text{TF}}, N_{\text{P}})$.

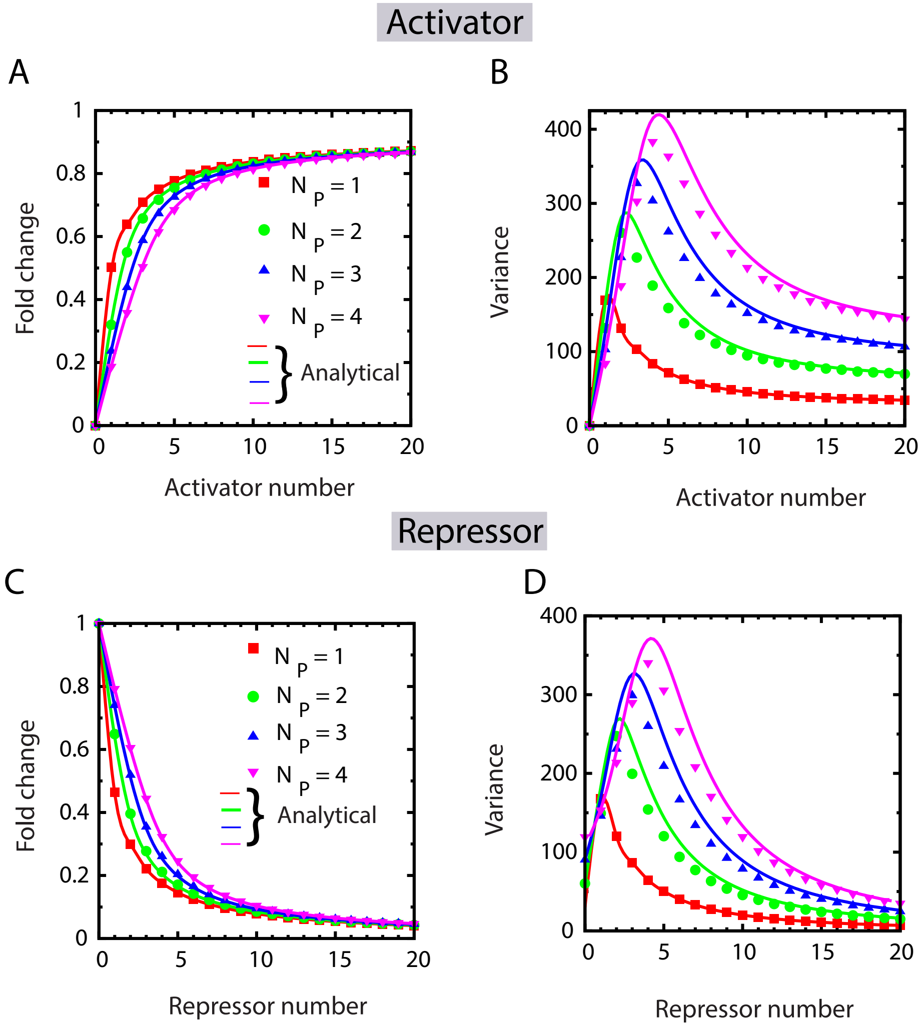

FIG. A: Analytical solutions predict the first two moments of the mRNA distribution. (A-B) Fold change (defined by $\langle m \rangle/(N_{\mathrm{P}} r/\gamma)$) and variance of the mRNA distribution for activators without any basal expression, as a function of the activator number. (C-D) Fold change and variance for repressors against the repressor number. In all the figures, solid continuous lines represent approximate analytical solutions, and symbols represent data from numerical simulations. The parameters are as specified in Table 1 within the main text.

The solutions of Eqs. 9-13 match extremely well at the mean level with the data from Gillespie simulations (see Fig. A); while the match is reasonably well at the variance level. In fact, the approximate analytical solutions clearly predict the peaks in variance as seen from the simulation (Fig. A). In Fig. B we show that the variance of complex number also exhibit a peak when the TF number equals the promoter number.

At the mean level, our model prediction is further in agreement with the bulk studies based on equilibrium statistical mechanics [1–3]. To show this, we plot the fold-change for repressors in Fig. C, which displays titration-like response when repressor number is tuned, as found in recent experiments [1] and theories [2, 3].

TFs acting as activators with a basal rate of expression

In the main text, we focused on two regulatory ubiquitous motifs of TFs in *E. coli*: (i) TFs as activators without any basal rate of expression, and (ii) TFs as repressors. These two cases can be obtained from our general model by setting (i) $r_{\text{free}} = 0, r_{\text{bound}} = r$, and (ii) $r_{\text{free}} = r, r_{\text{bound}} = 0$ respectively. However, we show in Fig. D that our results at mean and variance level still qualitatively hold when we incorporate a non-zero basal rate of transcription ($r_{\text{free}} = r_{\text{bound}}/20$). Importantly, the general prediction that the mRNA variance peaks when the TF number equals

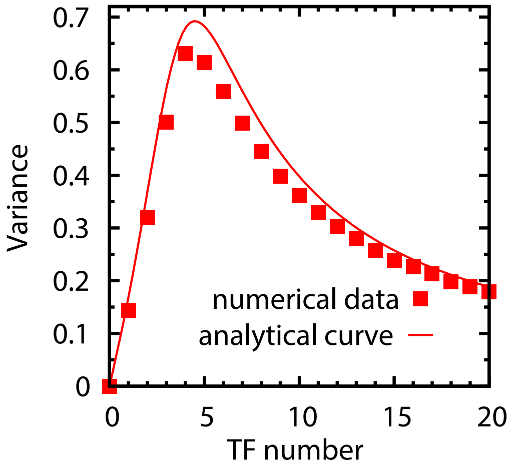

FIG. B: The variance of the complex number distribution for four promoter copies ($N_{\rm P} = 4$) as a function of the TF number. The solid continuous line represent approximate analytical solution, and the symbols represent data from simulations. The parameters are taken from Table 1 in the main text.

the promoter number still holds true.

Exact solutions for the special case of one promoter

As noted before, the exact closed-form expressions for the moments cannot be obtained since the equations for do not close. However, for the special case of one promoter ($N_{\rm P} = 1$), we can obtain exact solutions. For $N_{\rm P} = 1$, the possible complex numbers are only 0 and 1. Thus we can write $\langle n\rangle = \langle n^2\rangle = \langle n^3\rangle$..., and $\langle nm\rangle = \langle n^2 m\rangle = \langle n^3 m\rangle$, etc. Due to this simplification, the system of equations (Eqs 9-13) gets closed. By solving the equations we obtain

$$\langle m\rangle = \frac{(r_{\rm bound} k_{\rm on} N_{\rm TF} + r_{\rm free} k_{\rm off})}{\gamma(k_{\rm on} N_{\rm TF} + k_{\rm off})} \tag{14}$$

$$\mathrm{var}(m) = \langle m\rangle \left[1 + \frac{k_{\rm off} k_{\rm on} N_{\rm TF}}{(k_{\rm on} N_{\rm TF} + k_{\rm off})} \frac{(r_{\rm bound} - r_{\rm free})^2}{(r_{\rm bound} k_{\rm on} N_{\rm TF} + r_{\rm free} k_{\rm off})(\gamma + k_{\rm on} N_{\rm TF} + k_{\rm off})}\right] \tag{15}$$

In the above expressions, if we put $r_{\rm free} = 0$ or $r_{\rm bound} = 0$, we get the expressions for activators without basal expression, and for repressors respectively. This leads to Eq. 2 in the main text.

An interesting observation from Eq. 14, 15 is that both the mean and the variance depend on the TF number only through an effective variable $k_{\rm on}^{\rm eff} = k_{\rm on} N_{\rm TF}$. Thus, if a single promoter share the TFs with other competitor sites, the effective variable $k_{\rm on}^{\rm eff}$ gets altered without changing any other parameters (such as $k_{\rm off}, r_{\rm free}, r_{\rm bound}, \gamma$). Therefore, as discussed in the main text, the variance as a function of the mean follows a master curve irrespective of the number of the competitor sites and their binding and unbinding rates to the TFs. This is shown in Fig. E. By eliminating $k_{\rm on}^{\rm eff}$ from both mean and variance, and expressing the variance in terms of the mean, we obtain the following general expression for the master curve

$$\mathrm{var}(m) = \langle m\rangle + \frac{(r_{\rm bound} - \langle m\rangle\gamma)^2 (r_{\rm free} - \langle m\rangle\gamma)}{\gamma[(k_{\rm off}(r_{\rm free} - r_{\rm bound}) + \gamma(\langle m\rangle\gamma - r_{\rm bound})]} \tag{16}$$

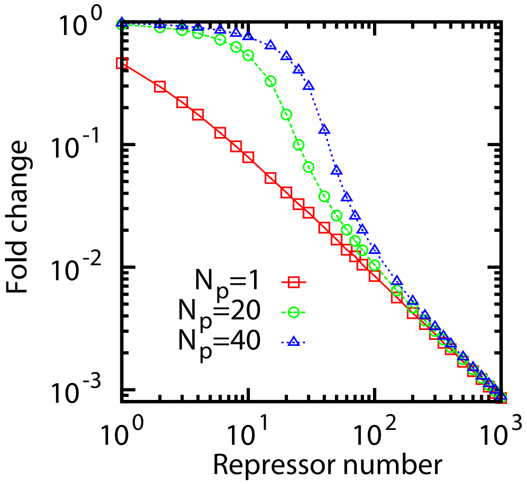

FIG. C: Log-log plot of the fold change in expression versus the repressor copy number. The parameters are specified in Table 1 within the main text.

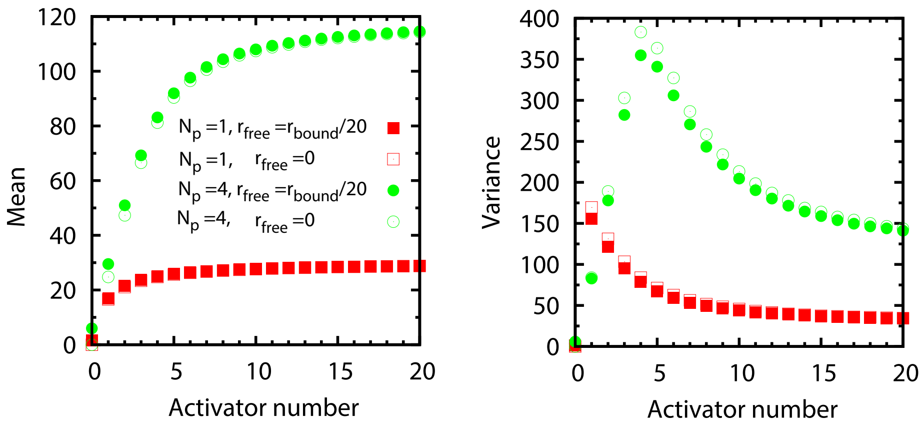

FIG. D: Comparison between the cases of activators without any basal expression, and with a basal expression level. Mean (left) and variance (right) of the mRNA distribution as a function of the activator number. The filled symbols represent the case of activators with a basal rate of transcription, while the open symbols represent activators without any basal expression. The basal transcription rate is $r_{\rm free} = r_{\rm bound}/20$ and $r_{\rm bound} = 0.33 s^{-1}$. Other parameters are taken from Table 1 in the main text.

Above expression further leads to the master curves for the specific cases of repressors and activators (without basal expression), as given in Eq. 3 within the main text.

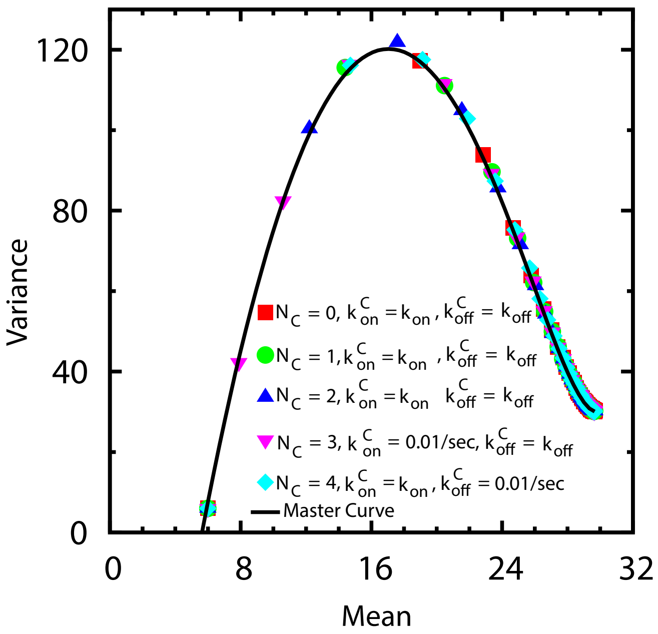

FIG. E: Variance as a function of mean when a single promoter share the TFs with other competitor binding sites. Here the TFs act as activators with a basal rate of expression ($r_{\rm free} = r_{\rm bound}/20$ as in Fig. D). The number of the competitor sites ($N_{\rm C}$) and its binding and unbinding rates to the TFs ($k^{\rm C}_{\rm on}$, $k^{\rm C}_{\rm off}$) are varied and listed in the figure, while other parameters are taken from Table 1 in the main text. The black continuous line is the analytical master curve taken from Eq. 16.

The special case of two promoters sharing TFs

For more than a single promoter, the calculation of the exact moments become challenging. However, we compute analytical expression of the mRNA mean for two promoters, to compare it with that of a single promoter. From Eq. 9, the mRNA mean for $N_{\rm P} = 2$ can be written as $\langle m \rangle = (\langle n \rangle r_{\rm bound} + (2 - \langle n \rangle) r_{\rm free})/\gamma$, where $\langle n \rangle$ is the mean complex number. It is straight forward to obtain the steady-state complex number distribution, $P(n)$ [4]. Thus for $N_{\rm P} = 2$, the mean complex number can be obtained easily as $\langle n \rangle = 1.P(1) + 2.P(2)$. Ultimately, we obtain the mean of the mRNA distribution, produced by the two promoter copies as below

$$\langle m \rangle|_{N_{\rm P}=2} = \frac{2[r_{\rm free} k^2_{\rm off} + (r_{\rm free} + r_{\rm bound}) k_{\rm on} k_{\rm off} N_{\rm TF} + r_{\rm bound} k^2_{\rm on} N_{\rm TF}(N_{\rm TF} - 1)]}{\gamma [k^2_{\rm off} + 2 k_{\rm on} k_{\rm off} N_{\rm TF} + k^2_{\rm on} N_{\rm TF}(N_{\rm TF} - 1)]} \qquad (17)$$

Note that the above expression cannot be written as a function of the effective variable $k_{\rm on} N_{\rm TF}$ (compare Eq. 14 and 17). In fact, nonlinear terms (like $k^2_{\rm on} N^2_{\rm TF}$) appear in the mean expression. Only for large number of TFs ($N_{\rm TF} \gg 1$), the expression in Eq. 17 reduces to $\langle m \rangle|_{N_{\rm P}=2} = 2 \langle m \rangle|_{N_{\rm P}=1}$, signifying that the mean expression of the two promoter copies is not always a simple sum of the individual means. In other words, the promoters are correlated. Thus, in the presence of competitor sites, the variance will not follow a master curve; rather it will bear a signature of the specific TF competition with the competitor sites.

Robustness of model prediction for identical competing promoters with respect to parameter variation

In the main text, we discussed three main predictions of mRNA noise for the scenario of multiple identical promoters competing for a pool of TFs. The predictions are: (i) The mRNA variance peaks when the TF number equals the promoter number for both activators and repressors. (ii) The Fano factor for activator also peaks when the TF number equals the promoter number. (iii) The Fano factor for repressor peaks when the TF number is slightly greater than (but comparable to) the promoter number. It is to be noted that there is no guarantee that both variance and Fano factor would show a peak at the same location. Nevertheless, we checked that for a wide range of parameter values our predictions hold true (see Fig. G-I).

There are some limiting conditions where the predictions are no longer true. For example, for very high TF binding rate (with respect to other parameters) all promoters are essentially always occupied. So the peaks disappear (see

Fig. I(A-C)). Similarly for very high TF unbinding rate, the promoters are always essentially unbound, and the peaks again disappear (Fig. H(A,F)). When the mRNA production rate is comparable (or smaller than) to mRNA decay rate, the mRNA number is always low and the peaks cannot be seen (Fig. G(A,C); H(E,J)). In summary, if the mRNA production/degradation kinetics dominates over the TF binding/unbinding kinetics, the peaks would not be visible.

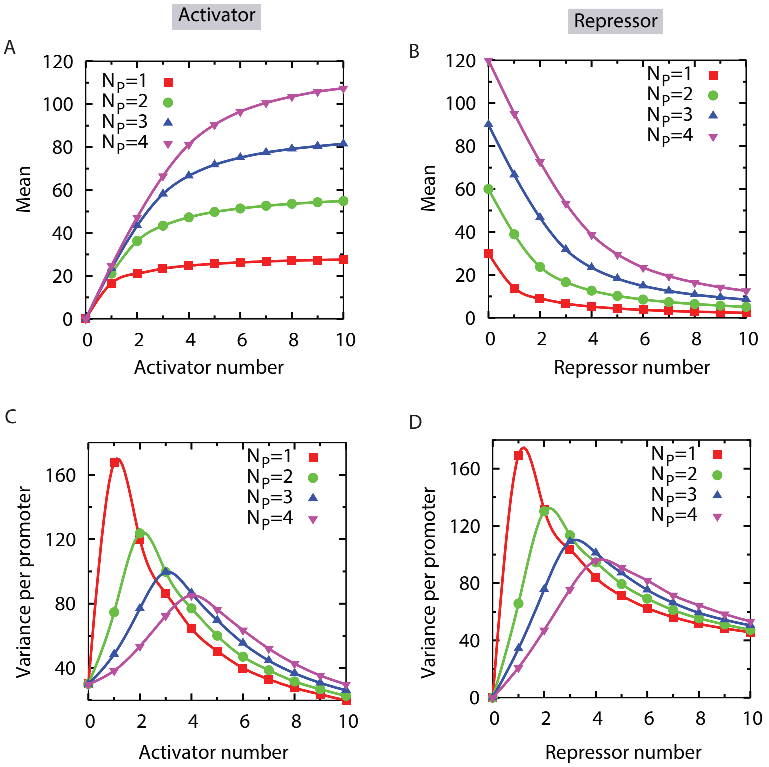

FIG. F: (A) Mean mRNA versus activator number. (B) Mean mRNA versus repressor number. (C) Variance of mRNA distribution per promoter versus activator number. (D) Variance per promoter versus repressor number. The parameters are taken from Table 1 in the main text.

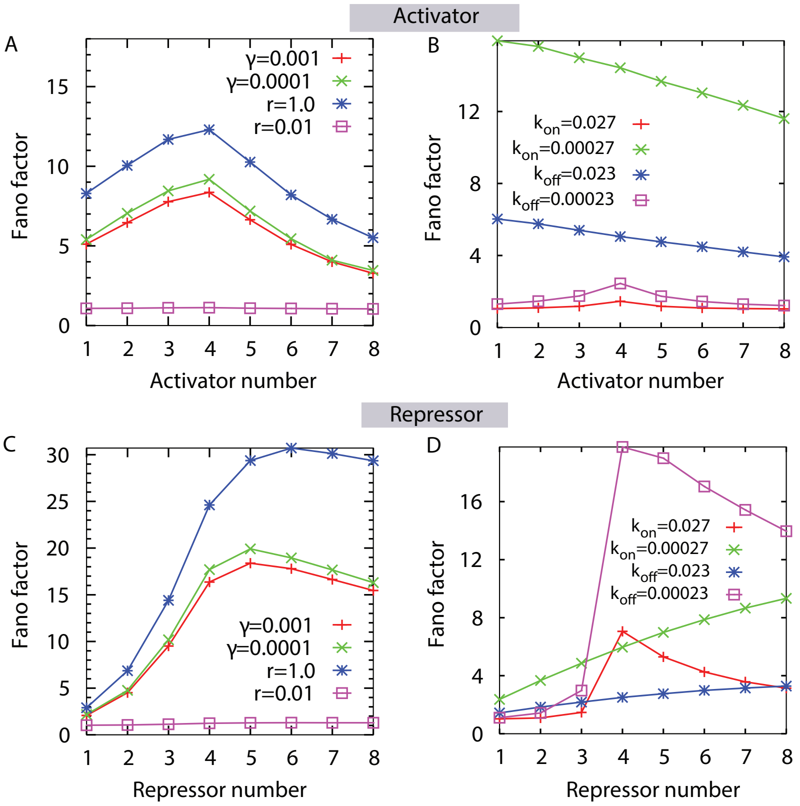

FIG. G: Robustness of Model prediction for the Fano factor of mRNA number. (A-B) Fano factor versus activator number (C-D) Fano factor versus repressor number. Each parameter that deviates from Table 1 (main text) is specified in the plots, while other parameters are taken from Table 1. The number of promoter copies is 4 in this case.

[1] Brewster RC, Weinert FM, Garcia HG, Song D, Rydenfelt M, Phillips R. The transcription factor titration effect dictates level of gene expression. Cell. 2014;156(6):1312–23.

[2] Rydenfelt M, Cox RS, Garcia H, Phillips R. Statistical mechanical model of coupled transcription from multiple promoters due to transcription factor titration. Phys Rev E. 2014;89:012702.

[3] Weinert FM, Brewster RC, Rydenfelt M, Phillips R, Kegel WK. Scaling of gene expression with transcription-factor fugacity. Phys Rev Lett. 2014;113(25):1–5.

[4] Firman T, Ghosh K. Competition enhances stochasticity in biochemical reactions. J Chem Phys. 2013;139(12).

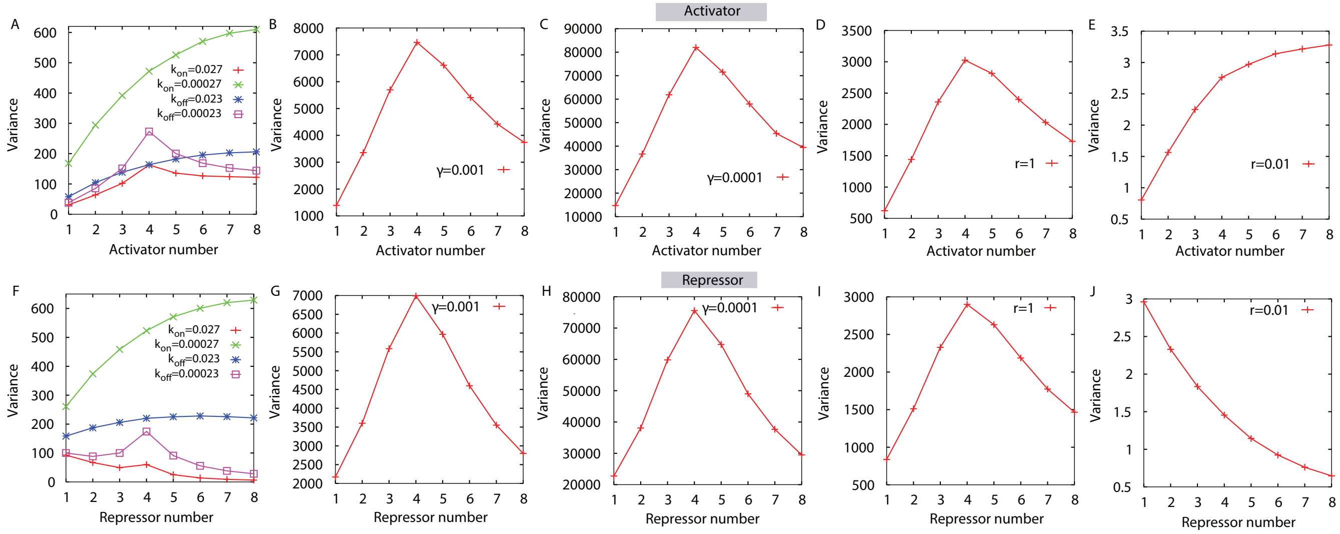

FIG. H: Robustness of Model prediction for mRNA variance. (A-E) Variance versus activator number (F-J) Variance versus repressor number. Each parameter that deviates from Table 1 (main text) is specified in the plots, while other parameters are taken from Table 1. The number of promoter copies is 4 in this case.

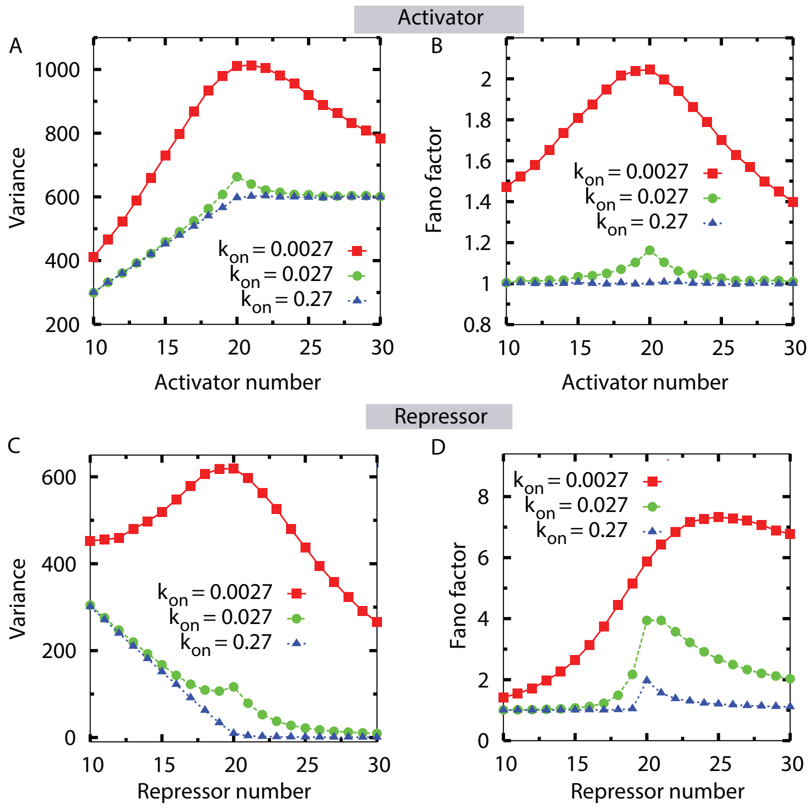

FIG. I: Robustness of Model predictions for large number of identical competing promoters. (A-B) Variance and Fano factor of mRNA number as functions of activator number (C-D) Variance and Fano factor of mRNA number as functions of repressor number. The TF binding rate to the promoters are varied and specified in the plots, while other parameters are taken from Table 1. The number of promoter copies is 20 in this case.

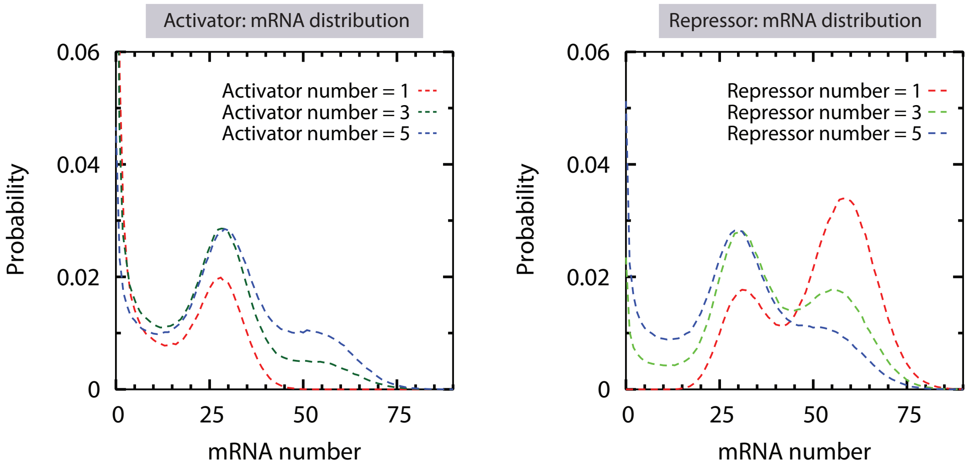

FIG. J: (A) Steady-state mRNA distribution for two identical promoters, sharing activators (left) and repressors (right). The TF binding rate to the promoters is much slower than specified in Table 1 ($k_{\rm on} = 0.0005$), while other parameters are taken from Table 1.

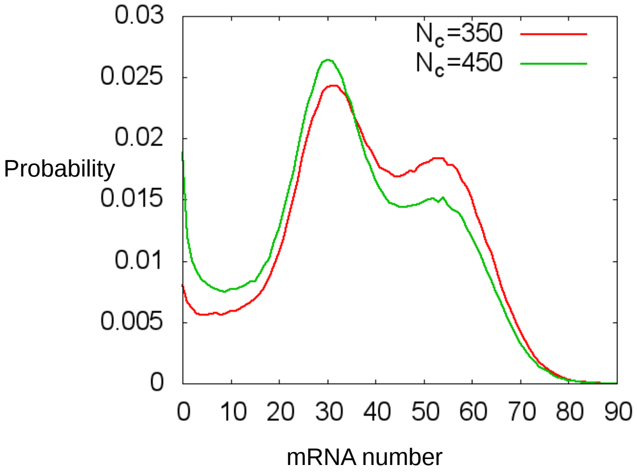

FIG. K: Steady-state mRNA distribution for two identical promoters sharing seven activators with a bunch of competitor sites. The activators bind to the competitor sites with a slower rate than to the promoters ($k_{\rm on} = 0.027, k_{\rm on}^C = 0.0027$), while they unbind from the competitor sites with a faster rate than from the promoters ($k_{\rm off} = 0.0023, k_{\rm on}^C = 0.023$). Multimodality arises if there are sufficient number of competitor sites. Similarly, multimodality can also be seen for the case of repressors (data not shown).

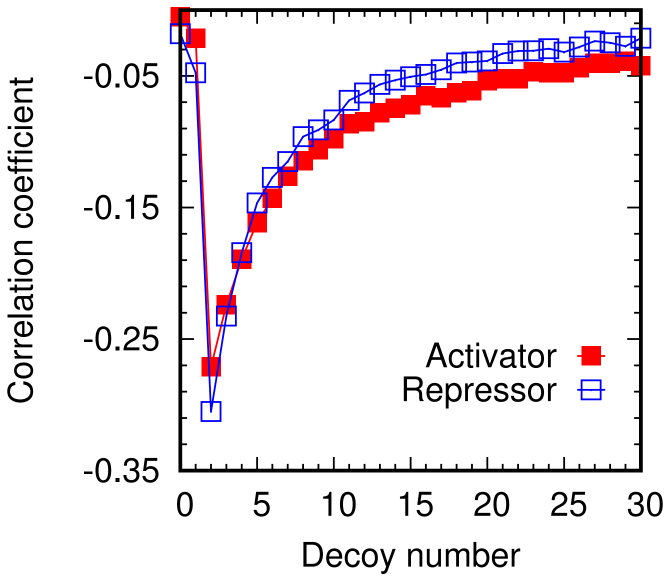

FIG. L: Pearson correlation coefficient between the mRNA molecules produced from two identical promoters in presence of several competitor sites. The plot is for correlation coefficient versus the number of competitor sites. The parameters are same as chosen for Fig. 5C,D in the main text.

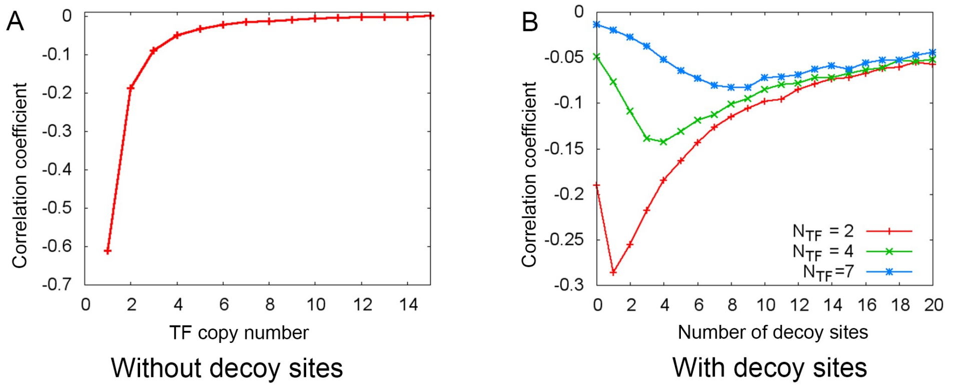

FIG. M: Pearson correlation coefficient between the mRNA molecules produced from two identical promoters in absence(A) and in presence (B) of several competitor sites. (A) Correlation coefficient versus the number of TF copy number. (B) Correlation coefficient versus the number of competitor sites for different fixed values of TF copy number. Note that the response with respect to the TF number variation, and competitor site number variation is distinct. The figure is for activators, but the same results hold for repressors as well (data not shown). The parameters are taken from Table 1.
